# Cytosolic nucleic acid sensors and interferon beta-1 activation drive radiation-induced anti-tumour immune effects in human pancreatic cancer cells

**DOI:** 10.3389/fimmu.2024.1286942

**Published:** 2024-09-20

**Authors:** Sylvia Kerschbaum-Gruber, Ava Kleinwächter, Katerina Popova, Alexandra Kneringer, Lisa-Marie Appel, Katharina Stasny, Anna Röhrer, Ana Beatriz Dias, Johannes Benedum, Lena Walch, Andreas Postl, Sandra Barna, Bernhard Kratzer, Winfried F. Pickl, Altuna Akalin, Filip Horvat, Vedran Franke, Joachim Widder, Dietmar Georg, Dea Slade

**Affiliations:** ^1^ Department of Radiation Oncology, Medical University of Vienna, Vienna, Austria; ^2^ Comprehensive Cancer Center, Medical University of Vienna, Vienna, Austria; ^3^ MedAustron Ion Therapy Center, Wiener Neustadt, Austria; ^4^ Max Perutz Labs, Vienna Biocenter Campus (VBC), Vienna, Austria; ^5^ Center for Medical Biochemistry, Medical University of Vienna, Vienna, Austria; ^6^ Vienna Biocenter PhD Program, a Doctoral School of the University of Vienna and the Medical University of Vienna, Vienna, Austria; ^7^ Center for Pathophysiology, Infectiology and Immunology, Institute of Immunology, Medical University of Vienna, Vienna, Austria; ^8^ Karl Landsteiner University of Health Sciences, Krems, Austria; ^9^ Max Delbrück Center, The Berlin Institute for Medical Systems Biology, Berlin, Germany

**Keywords:** interferon, radiation, protons, pancreatic cancer, STING, MAVS, NF-κB

## Abstract

**Introduction:**

Pancreatic ductal adenocarcinoma (PDAC) remains a leading cause of cancer-related deaths worldwide with limited treatment options due to extensive radiation and chemotherapy resistance. Monotherapy with immune checkpoint blockade showed no survival benefit. A combination of immunomodulation and radiotherapy may offer new treatment strategies, as demonstrated for non-small cell lung cancer. Radiation-induced anti-tumour immunity is mediated through cytosolic nucleic acid sensing pathways that drive the expression of interferon beta-1 (IFNB1) and proinflammatory cytokines.

**Methods:**

Human PDAC cell lines (PANC-1, MIA PaCa-2, BxPC-3) were treated with X-rays and protons. Immunogenic cell death was measured based on HMGB1 release. Cytosolic dsDNA and dsRNA were analysed by immunofluorescence microscopy. Cell cycle progression, MHC-I and PD-L1 expression were determined by flow cytometry. Galectin-1 and IFNB1 were measured by ELISA. The expression levels and the phosphorylation status of the cGAS/STING and RIG-I/MAVS signalling pathways were analysed by western blotting, the expression of *IFNB1* and proinflammatory cytokines was determined by RT-qPCR and genome-wide by RNA-seq. CRISPR-Cas9 knock-outs and inhibitors were used to elucidate the relevance of STING, MAVS and NF-κB for radiation-induced IFNB1 activation.

**Results:**

We demonstrate that a clinically relevant X-ray hypofractionation regimen (3x8 Gy) induces immunogenic cell death and activates IFNB1 and proinflammatory cytokines. Fractionated radiation induces G2/M arrest and accumulation of cytosolic DNA in PDAC cells, which partly originates from mitochondria. RNA-seq analysis shows a global upregulation of type I interferon response and NF-κB signalling in PDAC cells following 3x8 Gy. Radiation-induced immunogenic response is regulated by STING, MAVS and NF-κB. In addition to immunostimulation, radiation also induces immunosuppressive galectin-1. No significant changes in MHC-I or PD-L1 expression were observed. Moreover, PDAC cell lines show similar radiation-induced immune effects when exposed to single-dose protons or photons.

**Conclusion:**

Our findings provide a rationale for combinatorial radiation-immunomodulatory treatment approaches in PDAC using conventional photon-based or proton beam radiotherapy.

## 1 Introduction

Pancreatic ductal adenocarcinoma (PDAC) is a disease with dismal prognosis, especially when locally advanced. PDAC has the lowest 5-year survival of any solid tumour of 12.8% (1). Treatment is exceedingly difficult; the majority of patients present with advanced, unresectable disease to whom the standard of care with combinational radio-chemotherapy confers limited benefit (2). Immunotherapy is an emerging treatment strategy considered the fourth pillar of cancer treatment; its success, however, is restricted to a few malignancies, excluding PDAC (3). Immune checkpoint blockade (ICB) failed to show clinical efficacy as monotherapy in PDAC (4), which can be at least partially attributed to its highly desmoplastic and immunosuppressive tumour microenvironment (TME). PDAC are largely immunologically cold, phenotypically devoid of effector CD4+ and CD8+ T cells and dominated by immunosuppressive immune cell populations (5, 6). Radiotherapy is being increasingly recognized for its ability to induce anti-tumour effects beyond cell killing. Multiple preclinical studies including preclinical PDAC models have demonstrated increased efficacy when radiotherapy and ICB are combined (7–12). Moreover, early clinical data demonstrated encouraging results when combining ICB with radiotherapy for PDAC treatment (13–16).

Radiation may drive an anti-tumour response through multiple mechanisms, including a pro-immunogenic cellular state and immunogenic cell death (ICD). This type of regulated cell death initiates cytotoxic T-cell mediated adaptive immunity through the release or cell surface presentation of damage-associated-molecular pattern (DAMP) (17). Mechanistically, radiation-induced anti-tumour immunity can be induced by the accumulation of DNA in the cytosol of irradiated cells, which activates the cGAMP synthase (cGAS)/stimulator of interferon genes (STING) pathway and leads to the production of type 1 interferons (IFN-1), specifically interferon-beta 1 (*IFNB1*) and interferon-stimulated genes (ISGs) such as *CXCL10* (18, 19). Radiation was also shown to induce the mitochondrial antiviral-signalling (MAVS) pathway through the RNA sensor retinoic acid-inducible gene I (RIG-I), which also results in IFN-1 production (20–22). Both pathways rely on transcription factors interferon regulatory factors 3 and 7 (IRF3/IRF7) or nuclear factor kappa-light-chain-enhancer of activated B cells (NF-κB). NF-κB can induce *CXCL10* in an IFN-1-independent manner and promotes the expression of pro-inflammatory cytokines such as interleukin 6 (*IL-6*) and tumour necrosis factor alpha (*TNF-α*) as part of its canonical function (23, 24). Together, *IFNB1*, ISGs and NF-κB signalling orchestrate the recruitment and activation of immune cells, thus creating an *in situ* vaccine (25). Accretive evidence for the cGAS/STING-mediated anti-tumour immunity has emerged for multiple malignancies including PDAC (26). PDAC clinical samples expressing both cGAS and STING show infiltration of cytotoxic T-cells and higher overall survival (27).

Different radiotherapy parameters may influence the induction of an anti-tumour immune response, including type of radiation, radiation dose, fraction size and time post-radiation (28, 29). Photon-based radiotherapy of PDAC is limited due to the proximity of several radiation-sensitive organs at risk to the tumour, which can be effectively spared with protons due to the inverted depth-dose profile and reduced lateral scatter (30). In addition, protons may also lead to a different biological radiation response that could be therapeutically beneficial. Only a limited number of studies have investigated differential immune responses to photons versus protons and none have investigated proton-mediated immune effects in PDAC (31).

Here we compared the effects of photon and proton therapy on the activation of anti-tumour immunogenicity markers including the nucleic acid sensing/IFNB1 axis and canonical NF-κB using multiple dose levels and a clinically relevant fractionation regimen. In addition, we examined markers of immunosuppressive TME such as galectin-1 (Gal-1), which was recently implicated in impaired immune surveillance during PDAC progression (32), programmed death-ligand 1 (PD-L1) as the most prominent immune checkpoint protein expressed by tumour cells, and major histocompatibility complex class I (MHC-I), which is necessary for antigen presentation and T-cell activation.

We show that hypofractionated radiation induces the release of an immunogenic cell death signal in human PDAC cell lines. Compared to single dose treatment, hypofractionation is superior in activating IFNB1 through cytosolic nucleic acid sensing via STING and MAVS pathways. We found that radiation induces the secretion of Gal-1 but no change in PD-L1 or MHC-I expression, and that protons yield similar effects compared to photons with respect to cell survival and immunogenicity. Our findings demonstrate that radiation can stimulate the key signalling axis of anti-tumour immunity in human PDAC cells, supporting the potential therapeutic benefit of combinatorial neoadjuvant radiotherapy followed by immunotherapy using conventional photon-based radiotherapy or proton beam therapy.

## 2 Results

### 2.1 Radiation elicits immunogenic cell death in human PDAC cell lines

**Figure 1:**
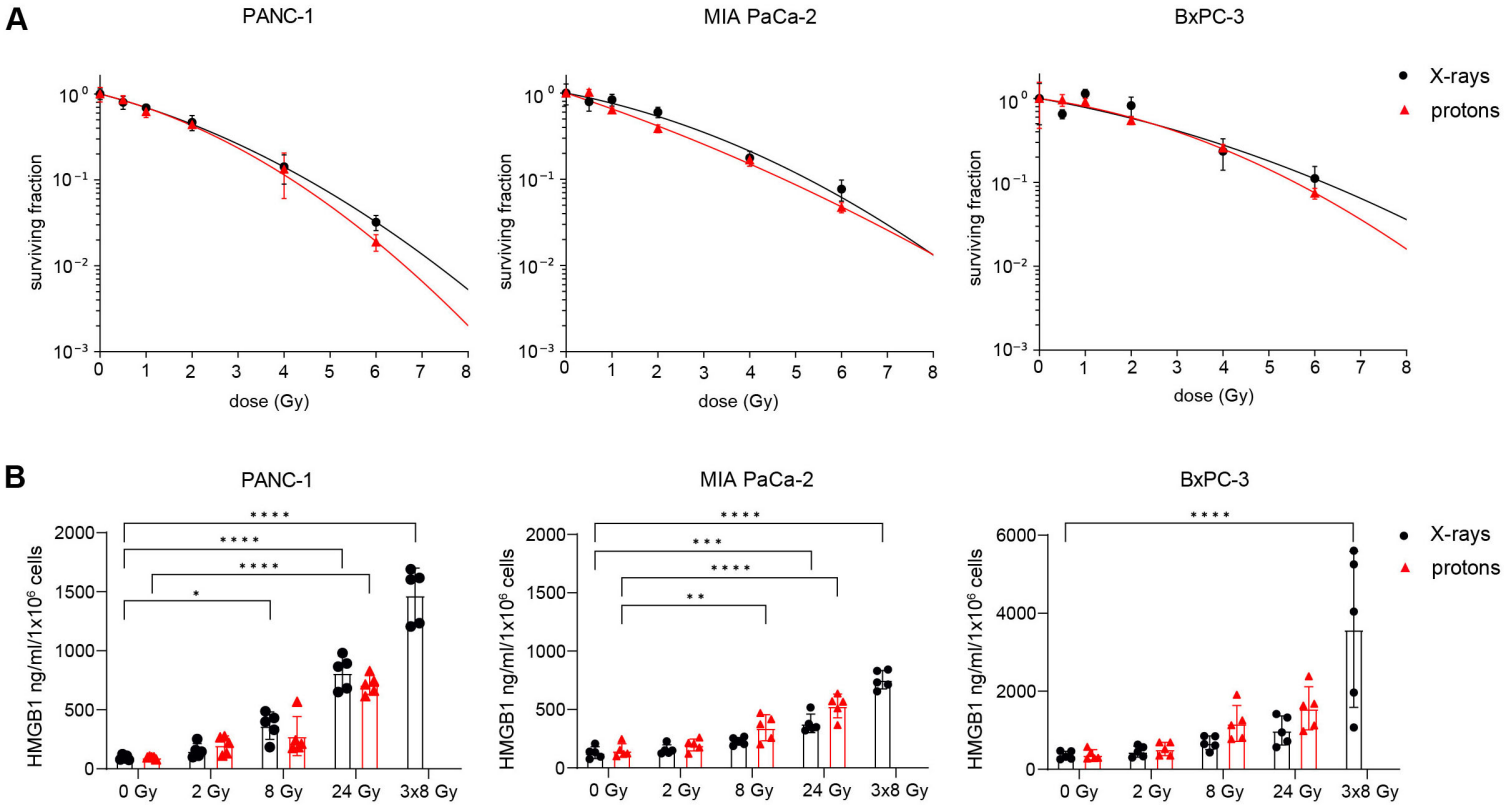
Radiation induces immunogenic cell death in human PDAC cell lines. **(A)** Cell survival curves of PANC-1, MIA PaCa-2 and BxPC-3 after reference X-ray (black circles) or proton irradiation (red triangles) (N=4-5). **(B)** Soluble HMGB1 was measured by ELISA in the supernatant 72 h post radiation (N=5). Data points represent mean values ± standard deviation. *≤ 0.05; **≤ 0.01; ***≤ 0.005, ****≤0.0001

Clonogenic survival was used to assess the effect of X-rays and protons on the viability of human PDAC cell lines PANC-1, MIA PaCa-2 and BxPC-3. All cell lines showed a dose-dependent decline of survival. RBE values were calculated at 10% cell survival (RBE_10_) from the applied linear-quadratic fit. For PANC-1, MIA PaCa-2 and BxPC-3, RBE_10_ values of 1.08 ± 0.02 Gy, 1.13 +/- 0.07 Gy and 1.11 ± 0.28 Gy were calculated, respectively (**Figure 1A**). To determine whether radiation-induced cancer cell death can elicit an anti-tumour immune response, we examined the release of HMGB1. HMGB1 acts as DAMP and is recognized by dendritic cells to elicit a potent tumour-specific T-cell response (33). Both irradiation modalities induced the ICD marker HMGB1 in a dose-dependent manner, while 3x8 Gy showed the strongest effect in all three cell lines. HMGB1 release kinetics were similar following X-rays or protons at equal physical doses (**Figure 1B**).

### 2.2 PDAC cells accumulate cytosolic dsDNA following irradiation

**Figure 2:**
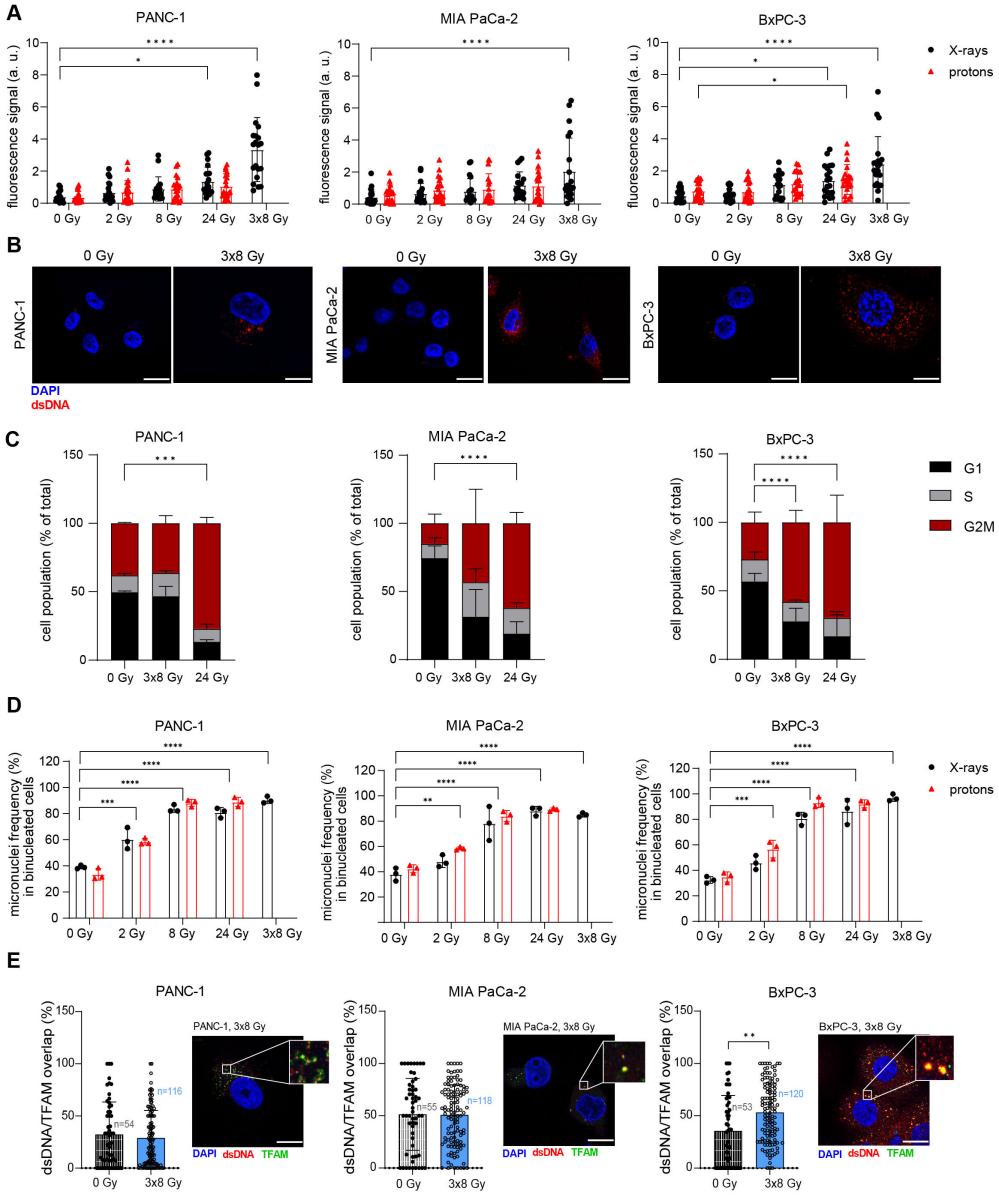
Radiation induces cytosolic dsDNA accumulation in human PDAC cell lines. **(A, B)** Cytosolic dsDNA content in PANC-1, MIA PaCa-2 and BxPC-3 cells 24 h post radiation with different doses of X-rays (black circles) or protons (red triangles). 5 cells were analysed per experiment, 4 independent experiments were performed. **(A)** Quantification and **(B)** representative images are shown. Scale bar = 20 µm. **(C)** Flow cytometry analysis of cell cycle distribution in untreated cells and 24 h after treatment with 3x8 Gy or 24 Gy X-rays (N = 4). **(D)** Percentage of micronuclei-positive PANC-1, MIA PaCa-2 and BxPC-3 cells exposed to different doses of X-rays. A cytokinesis block was induced with cytochalasin B 48 h after radiation for 24 h. Micronuclei-positive binucleated cells were scored. A minimum of 50 cells were analysed per experiment (N=3). **(E)** Colocalization analyses of dsDNA and TFAM in untreated cells and 24 h after treatment with 3x8 Gy X-rays using Airyscan high resolution imaging (N=3). Quantification and representative images are shown. Scale bar = 20 µm. Data points represent mean values ± standard deviation. *≤ 0.05; **≤ 0.01; ***≤ 0.005, ****≤0.0001.

Radiation-induced activation of IFNB1, which is crucial for radiation-induced anti-tumour immunogenicity, can be a consequence of cytosolic dsDNA or dsRNA accumulation. Thus, we tested whether radiation can cause cytosolic dsDNA and dsRNA accumulation in human PDAC cells using immunofluorescence microscopy. We observed a significant accumulation of cytosolic dsDNA after 3x8 Gy X-rays in all three cell lines (**Figures 2A, B**; **Supplementary Figure 1**). Only BxPC-3 showed an increase in cytosolic dsRNA after 8 Gy X-rays (**Supplementary Figure 2**).

Radiation-induced immunostimulatory DNA may originate from micronuclei (34) or damaged mitochondria (35, 36). Micronuclei arise from mitotic defects in chromosome segregation, whereby lagging chromosomes are encapsulated by their own nuclear envelope. Cell cycle analyses revealed a G2/M arrest after 24 Gy or 3x8 Gy X-rays in all three PDAC cell lines (**Figure 2C**). The cytokinesis block assay (37) showed a pronounced increase in micronuclei in all three cell lines after 8 Gy, 24 Gy and 3x8 Gy, which was comparable for the same dose of photons and protons (**Figure 2D**). To evaluate the mitochondrial origin of cytosolic dsDNA, we performed a co-localization analysis with the mitochondrial transcription factor A (TFAM) and found radiation-induced increase in dsDNA/TFAM co-localization in BxPC-3 24 h after exposure to 3x8 Gy X-rays (**Figure 2E**). Subcellular fractionation followed by qPCR analysis of mitochondrial DNA (mtDNA) and genomic DNA (gDNA) in the cytosolic fraction showed an increase in gDNA for all cell lines and an increase in mtDNA for PANC-1 and MIA PaCa-2 on Day 3 after exposure to 3x8 Gy X-rays (**Supplementary Figure 3**). Overall, our results show that 3x8 Gy X-rays triggers cytosolic dsDNA accumulation, which can act as an immunostimulatory signal originating from micronuclei or mitochondria.

### 2.3 A hypofractionation regimen of 3x8 Gy X-rays induces the type I interferon and NF-κB response in PDAC cell lines

**Figure 3:**
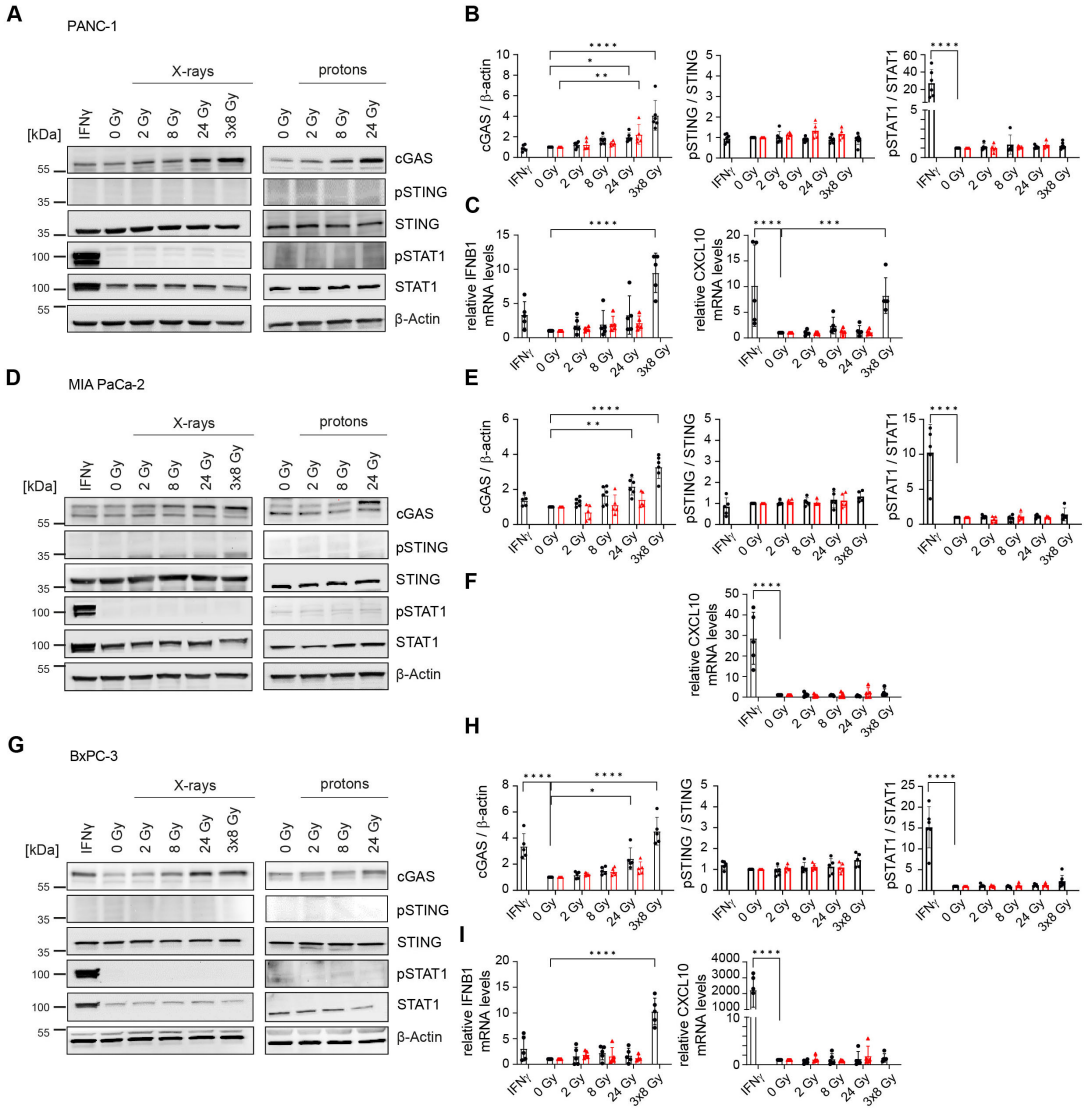
Hypofractionated irradiation (3x8 Gy) activates *IFNB1* in human PDAC cells. Activation of the cGAS/STING pathway 24 h after X-ray (black circles) or proton (red triangles) irradiation was analysed by western blotting and RT-qPCR in **(A-C)** PANC-1, **(D-F)** MIA PaCa-2 and **(G-I)** BxPC-3. **(A, D, G)** Representative western blots and **(B, E, H)** quantification based on five replicates are shown. GAPDH was used as a loading control. **(C, F, I)** Relative expression of *IFNB1* and the interferon-stimulated gene (ISG) *CXCL10* compared to 0 Gy was determined by RT-qPCR (N=5). No qPCR Ct values could be determined for MIA PaCa-2 *IFNB1*. Data points represent mean values ± standard deviation. *≤ 0.05; **≤ 0.01; ***≤ 0.005, ****≤0.0001.

To probe whether radiation-induced dsDNA accumulation can activate the cGAS/STING signal transduction pathway and IFN-1 response, as previously shown in lung, colorectal and cervical cancer (19, 38, 39), we investigated the effects of different irradiation regimens on the signalling cascade 24 h after radiation exposure. Radiation induced cGAS dose-dependently in all three cell lines following X-rays or protons. No radiation-mediated changes were observed for the other proteins. The hypofractionation regimen of 3x8 Gy, but not the single doses, increased the expression of *IFNB1* in PANC-1 and BxPC-3 (**Figure 3**). In MIA PaCa-2, *IFNB1* could not be detected in any experimental condition. Upon release into extracellular space, IFNB1 binds to interferon-α/β receptor (IFNAR) on neighbouring cells, resulting in phosphorylation of STAT1. All three investigated cell lines express IFNAR (40). STAT1 phosphorylation was not observed 24 h post-irradiation in any of the three cell lines, despite significantly increased *IFNB1* transcripts in PANC-1 and BxPC-3 (**Figure 3**). Interferon-gamma (IFNγ) was used as a positive control, demonstrating intact STAT1 phosphorylation capacity in all three cell lines.

**Figure 4:**
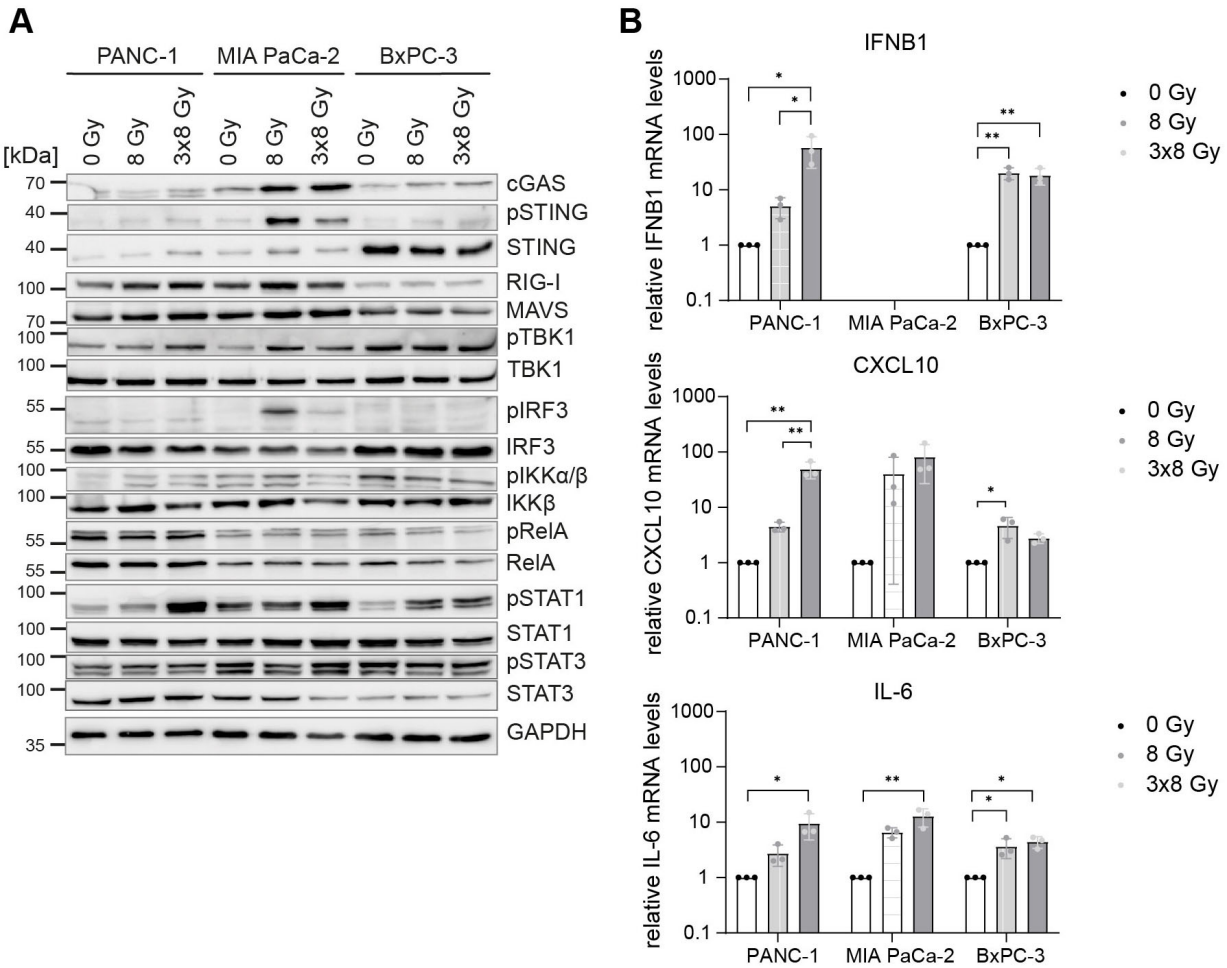
Comparative expression levels and activation of cGAS/STING, RIG-I/MAVS, NF-κB and STAT proteins in untreated and irradiated PDAC cells. PANC-1, MIA PaCa-2 and BxPC-3 cells were untreated or irradiated with 8 Gy or 3x8 Gy X-rays and harvested 72 h after radiation. **(A)** Representative western blots and **(B)** RT-qPCR analysis of *IFNB1*, *CXCL10* and *IL-6* compared to 0 Gy. Data points represent mean values of 5 independent experiments ± standard deviation. *≤ 0.05; **≤ 0.01.

Given that *IFNB1* gene expression was induced, we hypothesized that IFN-1 signalling and STAT1 phosphorylation are delayed in irradiated PDAC cells. To test this, we performed a time course analysis of all doses of X-rays (**Supplementary Figures 4**, **5**). Indeed, STAT1 phosphorylation, which is indicative of extracellular IFNB1 presence and thus active paracrine IFN-1 signalling, was observed from day 2 onwards after exposure to 3x8 Gy X-rays (**Supplementary Figure 4**). The increase in pSTAT1 was coupled with the induction of *IFNB1* and the interferon-stimulated gene *CXCL10* in PANC-1 and BxPC-3, whereas MIA PaCa-2 only showed *CXCL10* induction (**Supplementary Figure 4**). Interestingly, a single dose of 8 Gy X-rays consistently showed the induction of *IFNB1* and/or *CXCL10* in all cell lines 4 days after irradiation (**Supplementary Figure 5**), suggesting that cell cycle progression is required for the activation of IFN-1 response (34, 41). Compared to 8 Gy single dose irradiation, the hypofractionation regimen of 3x8 Gy was more potent in inducing *IFNB1* and/or *CXCL10* expression (**Figure 4**).

Of the three PDAC cell lines, PANC-1 mounted the strongest IFN-1 response to 3x8 Gy X-rays as evidenced by the induction of pSTAT1, *IFNB1* and *CXCL10* (**Figure 4**). PANC-1 also showed the highest expression of RIG-I/MAVS and the canonical NF-κB subunit RelA (p65) (**Figure 4A**). Conversely, STING and IRF3 levels were the highest in BxPC-3 (**Figure 4A**). Despite a strong induction of pSTING and pIRF3 in MIA PaCa-2, this cell line failed to induce *IFNB1* expression after radiation (**Figure 4**).

**Figure 5:**
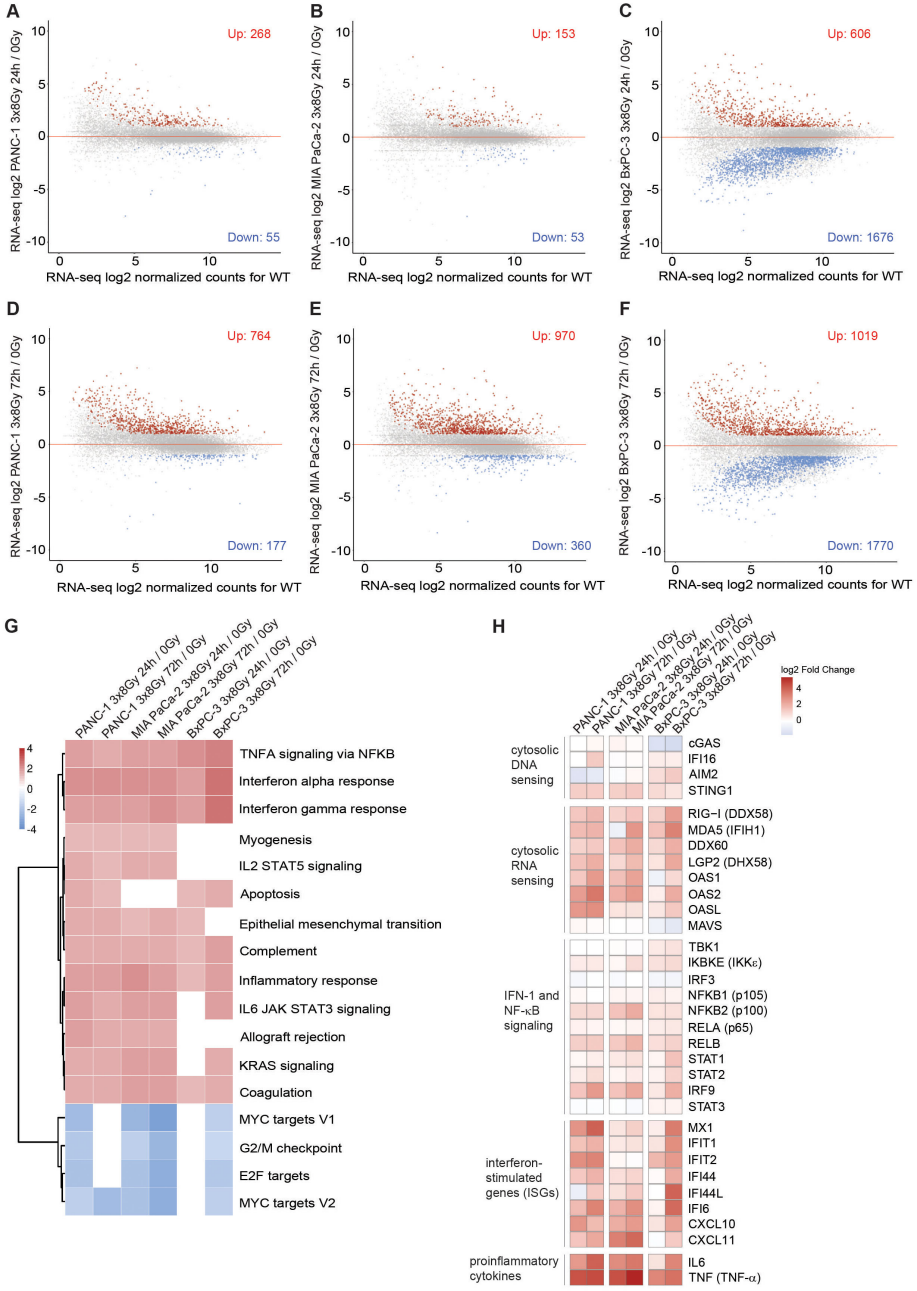
Type I interferon response and NF-κB signalling are globally upregulated in human PDAC cells after fractionated X-ray dose. **(A-C)** RNA-seq analysis in **(A)** PANC-1, **(B)** MIA PaCa-2 and **(C)** BxPC-3 cells 24 h after 3x8 Gy of X-rays compared to untreated. **(D-F)** RNA-seq analysis in **(D)** PANC-1, **(E)** MIA PaCa-2 and **(F)** BxPC-3 **(A)** PANC-1, **(B)** MIA PaCa-2 and **(C)** BxPC-3 cells 72 h after 3x8 Gy of X-rays compared to untreated. Genes with fold-change>2, p<0.05 are marked in red as upregulated or blue as downregulated (N=3). **(G)** GO analysis of upregulated genes shows enrichment of genes involved in type I interferon response and NF-κB signalling. Normalized enrichment scores (NESs) for Hallmark gene sets (MSigDB) between indicated irradiation treatments and controls in PANC-1, MIA PaCa-2 and BxPC-3 cells. Shown are pathways significant in at least 4 comparisons with significance level p < 0.05 adjusted for multiple testing. **(H)** Log2 fold changes in gene expression between indicated irradiation and control samples of chosen genes.

To examine radiation-induced changes in gene expression genome-wide, we performed RNA-seq in untreated cells and on day 1 and day 3 after 3x8 Gy (**Figures 5A-F**). We found upregulation of >700 genes (fold change >2; p-value <0.05) in all three PDAC cell lines on Day 3 after radiation exposure. Gene ontology (GO) analysis showed enrichment of genes implicated in IFN-1 signalling and canonical NF-κB signalling (**Figure 5G**). Genes involved in proliferation (MYC and E2F targets) and G2/M checkpoint were enriched among downregulated genes (**Figure 5G**). Among highly upregulated genes we identified many genes implicated in cytosolic nucleic acid sensing, non-canonical NF-κB complex (NFKB2/RELB), STAT1-STAT2-IRF9 (ISGF3 complex), ISGs and inflammatory cytokines (**Figure 5H**). Overall, our data show that radiation induces genome-wide IFN-1 and NF-κB response in human PDAC cells.

### 2.4 Radiation-induced type I interferon response is STING- and MAVS-dependent

**Figure 6:**
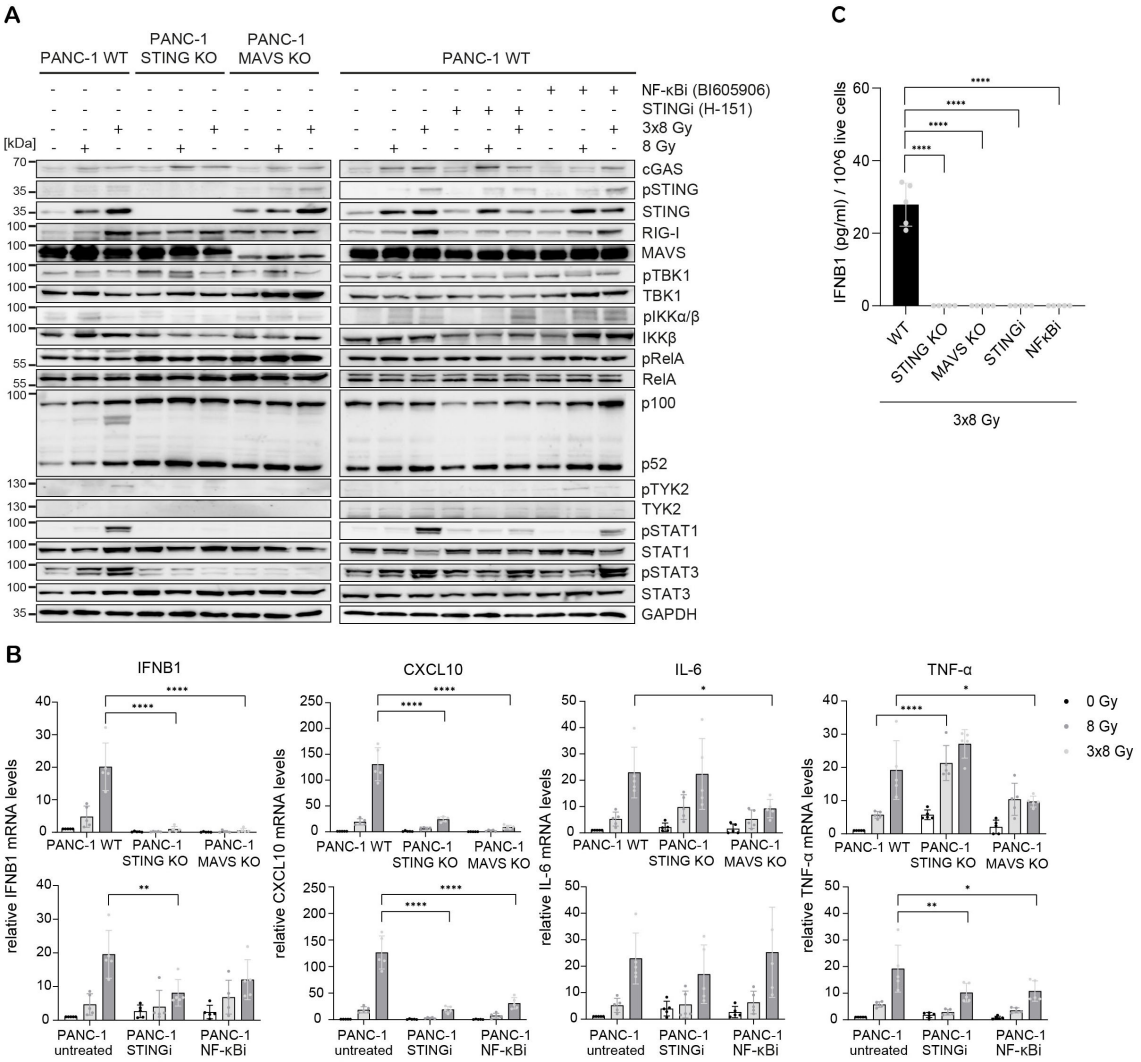
STING, MAVS and NF-κB drive radiation-induced immunogenic signalling in PANC-1. PANC-1 WT, STING KO and MAVS KO cells were untreated (0 Gy) or irradiated with 8 Gy or 3x8 Gy X-rays and harvested 72 h after radiation. WT cells were also treated with STINGi (10 µM H-151) or NF-κBi (10 µM BI-605906) without or with radiation. Cells were pretreated with inhibitors for 1 h before irradiation and kept until harvesting. **(A)** Representative western blot analysis of cGAS/STING, RIG-I/MAVS, IFN-1 and NF-κB signalling. **(B)** Relative mRNA levels of *IFNB1*, *CXCL10, IL-6* and *TNF-α* compared to untreated WT cells. **(C)** IFNB1 was measured in the supernatant of PANC-1 cells by ELISA. Data points represent mean values of 5 independent experiments ± standard deviation. *≤ 0.05; **≤ 0.01; ***≤ 0.005.

**Figure 7:**
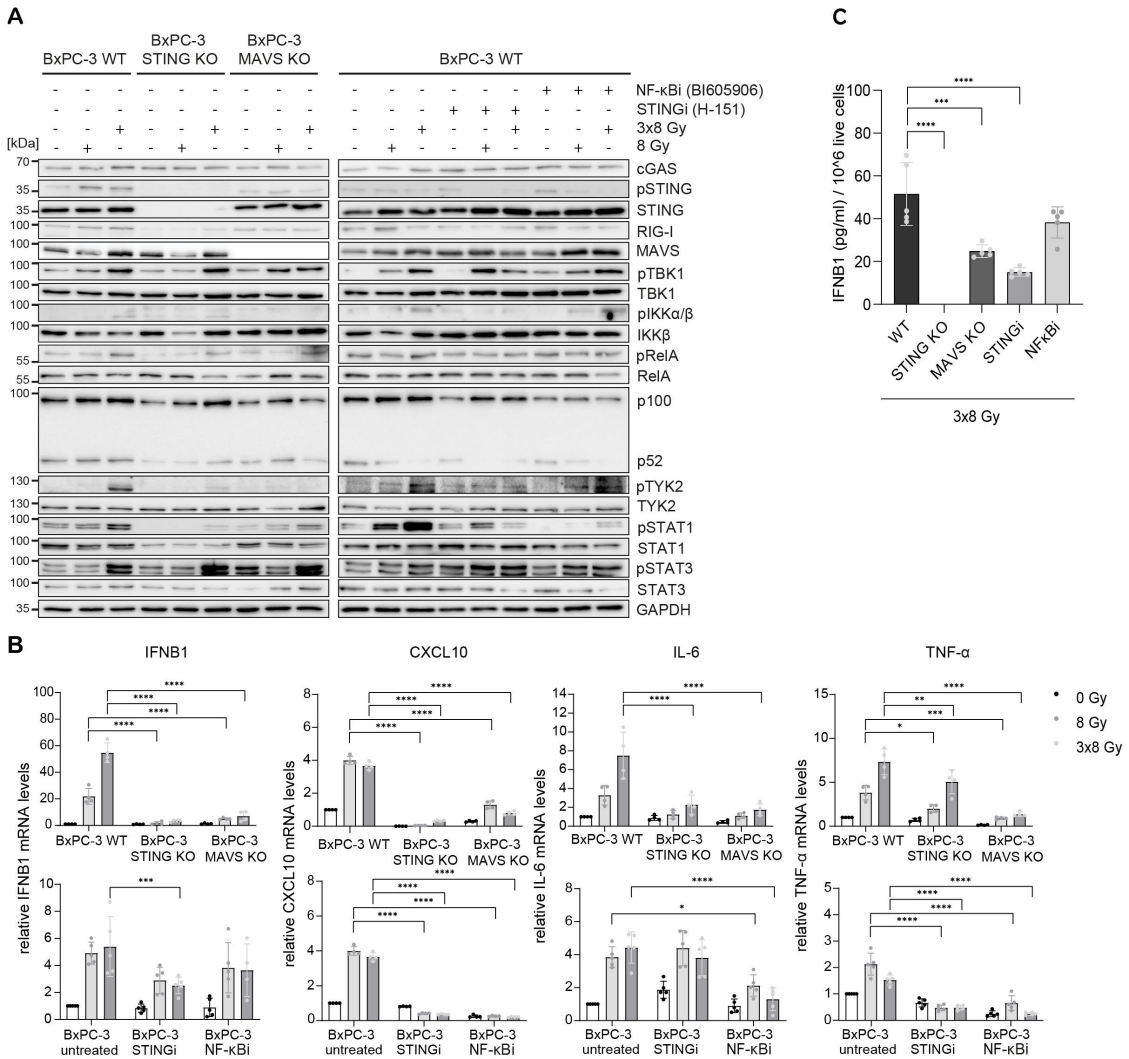
STING, MAVS and NF-κB drive radiation-induced immunogenic signalling in BxPC-3. BxPC-3 WT, STING KO and MAVS KO cells were untreated (0 Gy) or irradiated with 8 Gy or 3x8 Gy X-rays and harvested 72 h after radiation. WT cells were also treated with STINGi (10 µM H-151) or NF-κBi (10 µM BI-605906) without or with radiation. Cells were pretreated with inhibitors for 1 h before irradiation and kept until harvesting. **(A)** Representative western blot analysis of cGAS/STING, RIG-I/MAVS, IFN-1 and NF-κB signalling. **(B)** Relative mRNA levels of *IFNB1*, *CXCL10, IL-6* and *TNF-α* compared to untreated WT cells. **(C)** IFNB1 was measured in the supernatant of BxPC-3 cells by ELISA. Data points represent mean values of 4-5 independent experiments ± standard deviation. *≤ 0.05; **≤ 0.01; ***≤ 0.005, ****≤0.0001.

**Figure 8:**
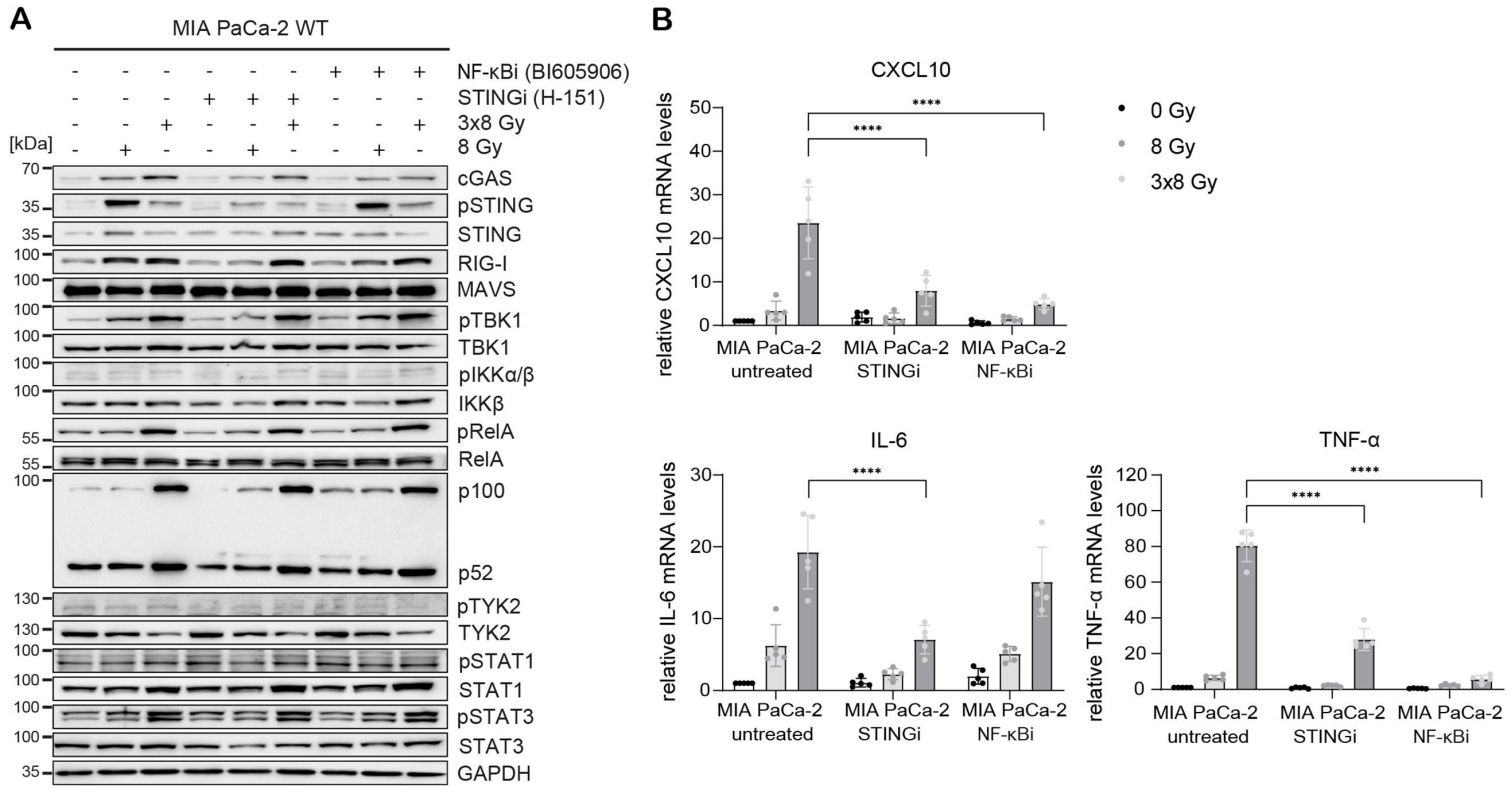
STING and NF-κB drive radiation-induced immunogenic signalling in MIA PaCa-2. MIA PaCa-2 cells were either untreated (0 Gy) or irradiated with 8 Gy or 3x8 Gy X-rays and harvested 72 h after radiation without or with STINGi (10 µM H-151) or NF-κBi (10 µM BI-605906). **(A)** Representative western blot analysis of cGAS/STING, RIG-I/MAVS, IFN-1 and NF-κB signalling. **(B)** Relative mRNA levels of *CXCL10, IL-6* and *TNF-α* compared to untreated WT cells. Data points represent mean values of 5 independent experiments ± standard deviation. ****≤0.0001.

Radiation-induced IFN-1 response was previously shown to be MAVS-dependent in PANC-1 cells (21). To test the relative requirement of STING and MAVS pathways for the induction of IFNB1 after radiation, we generated CRISPR/Cas9 knock-outs (KOs) of STING and MAVS in PANC-1 and BxPC-3 cells and tested their effect on the induction of *IFNB1* on day 3 after 8 Gy and 3x8 Gy X-ray radiation (**Figures 6**, **7**). In all three cell lines we additionally compared the effect of the STING inhibitor (H-151) (42) and the inhibitor of NF-κB signalling (BI605906) (43), as this pathway was also induced after radiation based on RNA-seq analysis (**Figure 5G**). BI605906 inhibits IKKβ and thereby prevents activation of RelA (p65) (43). STING and MAVS KO abrogated pSTAT1, *IFNB1* and *CXCL10* induction in PANC-1 (**Figure 6**) and BxPC-3 (**Figure 7**) and IFNB1 protein levels in the supernatant as measured by ELISA (**Figures 6C**, **7C**). Phosphorylation of TYK2, as a marker of IFNAR activation, was also abrogated in STING and MAVS KO cells (**Figures 6A**, **7A**). STING KO showed a slightly stronger effect in BxPC-3, in line with a higher STING expression in this cell line, whereas MAVS KO showed a stronger effect in PANC-1 cells, which express higher RIG-I/MAVS levels (**Figures 4**, **6**, **7**). The importance of STING for radiation-induced IFN-1 response was corroborated by siRNA-mediated STING depletion in PANC-1 and BxPC-3 treated with 3x8 Gy X-rays (**Supplementary Figure 6**). STING depletion resulted in diminished phosphorylation of STAT1 (**Supplementary Figures 6A, C**) and diminished induction of *IFNB1* and *CXCL10* (**Supplementary Figures 6B, D**) compared to non-targeting siRNA controls. STING siRNA depletion reduced, but did not abrogate radiation-induced IFN-1 response most likely due to partial STING silencing. Similarly, STING inhibition reduced, but did not abrogate, *IFNB1* and *CXCL10* activation in PANC-1 and BxPC-3 (**Figures 6**, **7**), and reduced *CXCL10* activation in MIA PaCa-2 (**Figure 8**).

NF-κB signalling was assessed based on the expression of proinflammatory cytokines IL-6 and TNF-α. In PANC-1 cells, IL-6 expression was reduced only in MAVS KO, whereas TNF-α expression was reduced in MAVS KO and NF-κB inhibitor-treated cells (**Figure 6**), suggesting that in PANC-1 cells IL-6 expression is partly regulated through the MAVS pathway independent of NF-κB, whereas TNF-α expression is regulated by both MAVS-dependent and MAVS-independent NF-κB signalling. Interestingly, the expression of RelA (canonical NF-κB) and p100/p52 (non-canonical NF-κB) was increased in PANC-1 STING and MAVS KO cells, which may act as a compensatory mechanism to sustain the expression of proinflammatory cytokines independent of STING and MAVS.

In BxPC-3 cells, the expression of IL-6 and TNF-α was reduced, but not abrogated, in STING and MAVS KOs and in cells treated with the NF-κB inhibitor (**Figure 7**). This suggests that radiation-induced expression of IL-6 and TNF-α is regulated by both STING/MAVS-dependent and independent NF-κB signalling in BxPC-3 cells.

In MIA PaCa-2 cells, radiation-induced expression of CXCL10, IL-6 and TNF-α was reduced after STING or NF-κB inhibition (**Figure 8**), suggesting that the expression of these cytokines is regulated by STING-dependent and STING-independent NF-κB signalling. Interestingly, the non-canonical NF-κB pathway was highly upregulated after radiation irrespective of STING or canonical NF-κB (**Figure 8A**). This may explain the lack of *IFNB1* expression in MIA PaCa-2, given that the non-canonical NF-κB pathway was shown to dampen *IFNB1* expression by epigenetically regulating its promoter (44).

Taken together, our data show that radiation-induced *IFNB1* expression is dependent on STING and MAVS pathways, whereas the expression of proinflammatory cytokines IL-6 and TNF-α is controlled by STING/MAVS-dependent and independent NF-κB signalling.

### 2.5 Radiation-mediated effects on T-cell modulating factors

**Figure 9:**
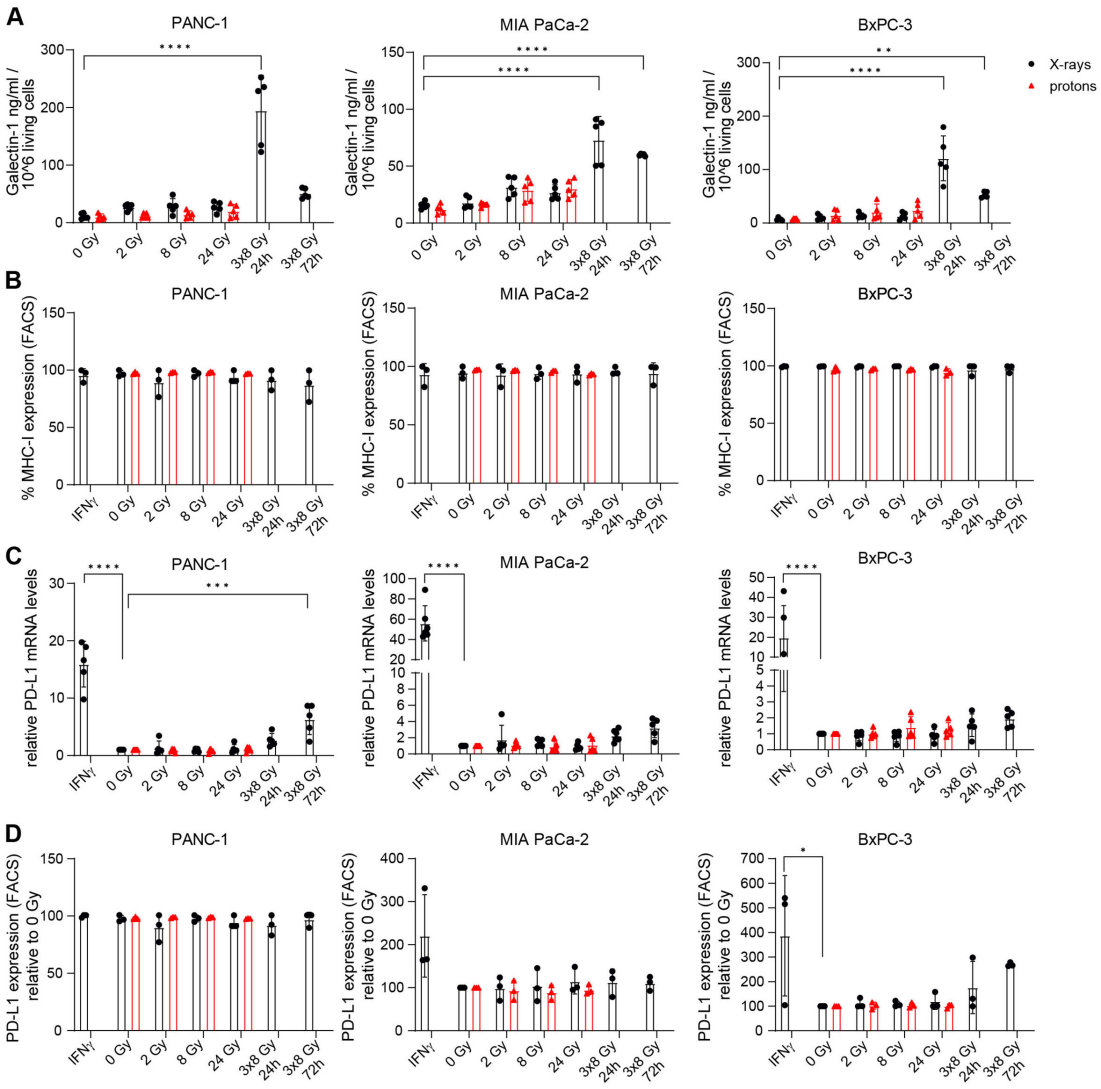
The effects of radiation on Galectin-1, MHC-I and PD-L1 in PDAC cells. **(A)** Galectin-1 was measured by ELISA in the supernatant of PDAC cells exposed to different doses of X-ray (black circles) or proton (red triangles) radiation 24 h post radiation and also 72 h after 3x8 Gy (N=5). **(B)** MHC-I expression was analysed by flow cytometry (N=3). **(C,D)** PD-L1 levels were measured by **(C)** RT-qPCR (N=5) and **(D)** FACS (N=3). Data points represent mean values of 3 to 5 independent experiments ± standard deviation. *≤ 0.05; **≤ 0.01; ***≤ 0.005, ****≤0.0001.

Modulating the TME is expected to improve response to immunotherapy for pancreatic cancer. Hypofractionation with 3x8 Gy was found to elicit the IFN-1 response that aims at immune effector recruitment, including cytotoxic T-cells. Successful T-cell activation depends on various factors, for which radiation-mediated effects could either be beneficial or deleterious in the context of anti-tumour efficacy. Thus we examined proteins with known immune effector interactions such as Gal-1, MHC-I and PD-L1. Gal-1 was implicated in the development and maintenance of an immunosuppressive TME by inducing helper- and cytotoxic T-cell apoptosis and anergy, promoting tolerogenic dendritic cells and expanding regulatory T-cells (33). Radiation-induced upregulation of Gal-1 was reported in human glioma cells (45) but no data is available for PDAC. We detected increased Gal-1 secretion at 24 h and 72 h after 3x8 Gy, but not after single dose irradiation (**Figure 9A**).

Cancer immune recognition is contingent on tumour peptide antigen presentation via MHC-I. Limited MHC-I expression in PDAC patients contributes to immunosuppressive TME by hindering tumour antigen presentation (46). Radiation-mediated stimulation of MHC-I expression was observed in murine pancreatic cancer models (47). However, MHC-I expression was not affected by radiation in our study (**Figure 9B**).

PD-L1 is an immune checkpoint protein that supresses antigen-independent T-cell activation and is often upregulated in cancers to evade immune surveillance. PD-L1 is commonly found in PDAC patients but PD-L1 blockade monotherapy failed in clinical studies due to the lack of intratumoral T-cells and the presence of myeloid suppressor cell populations. Radiation-mediated upregulation of PD-L1 cell surface expression was described in murine PDAC cell lines (8, 48). For human cell lines, no data is available and thus we assessed the effect of radiation on PD-L1 expression. *PD-L1* expression was induced after IFNγ treatment in all three cell lines and at 72 h after 3x8 Gy in PANC-1 (**Figure 9C**). IFNγ-mediated PD-L1 stimulation was confirmed on the protein level in BxPC-3 (**Figure 9D**). Radiation with either X-rays or protons did not affect PD-L1 protein levels in any of the three investigated PDAC cell lines (**Figure 9D**).

## 3 Discussion

The majority of pancreatic cancer patients did not benefit from recent advances in cancer immunotherapy due to immunosuppressive TME (4). Relapse affects up to 80% of pancreatic cancer patients within a few months after therapy (49). Remodelling the immunosuppressive TME by enhancing T-cell priming and preventing T-cell exhaustion may be the only road to success for this early metastasizing malignancy that has proven highly refractory to any treatment. Radiotherapy can shift immunologically unresponsive tumours into an immunologically more favourable phenotype by inducing an anti-tumour innate immune response through IFN-1 signalling (19, 38, 39). By converting tumours into an *in situ* vaccine, radiotherapy could activate an organism-wide tumour control mechanism and establish anti-tumour immune memory to prevent relapse. The likelihood of achieving immune-mediated disease control was shown to depend on optimal radiation dose/fractionation regimen (26) and may vary between malignancy types.

In this study, we assessed a variety of immunogenic radiation response parameters in three human PDAC cell lines. We used photon and proton single doses and a clinically relevant photon hypofractionation scheme to decipher whether (i) PDAC cells undergo immunogenic alterations in response to radiation, (ii) a specific radiation dose or fractionation regimen is more immunostimulatory, (iii) IFN-1 response is temporally modulated, and (iv) protons yield differential responses compared to photons at equal physical doses. We investigated the anti-tumour immunity conferred by the STING/MAVS/IFN-1 axis in a multifaceted approach and provide evidence for radiation-induced immunogenic cell death and immunogenicity in PDAC cells exposed to a hypofractionation radiation regimen of 3x8 Gy. We found that protons are slightly more effective compared to photons with RBE_10_ values around 1.1 for all three investigated cell lines, which supports the continued use of a uniform RBE assuming 10% increased effectiveness in the clinical setting.

We furthermore show that hypofractionated radiation treatment elicits accumulation of cytosolic dsDNA, phosphorylation of STING and STAT1, and induction of *IFNB1* and ISG expression in a time-dependent manner. In line with previous reports (34, 41), radiation-induced IFN-1 response is delayed and most likely dependent on mitotic progression and accumulation of DNA damage. Mitochondrial DNA was reported as a significant contributing factor in mouse mammary carcinoma cells and may also play a role in the context of PDAC, based on the observed co-localization of cytosolic DNA with the mitochondrial transcription factor TFAM and assessment of cytosolic mitochondrial DNA via qPCR. Although micronuclei accumulate after irradiation, recent studies show that micronuclei do not activate cGAS/STING (50, 51).

**Figure 10:**
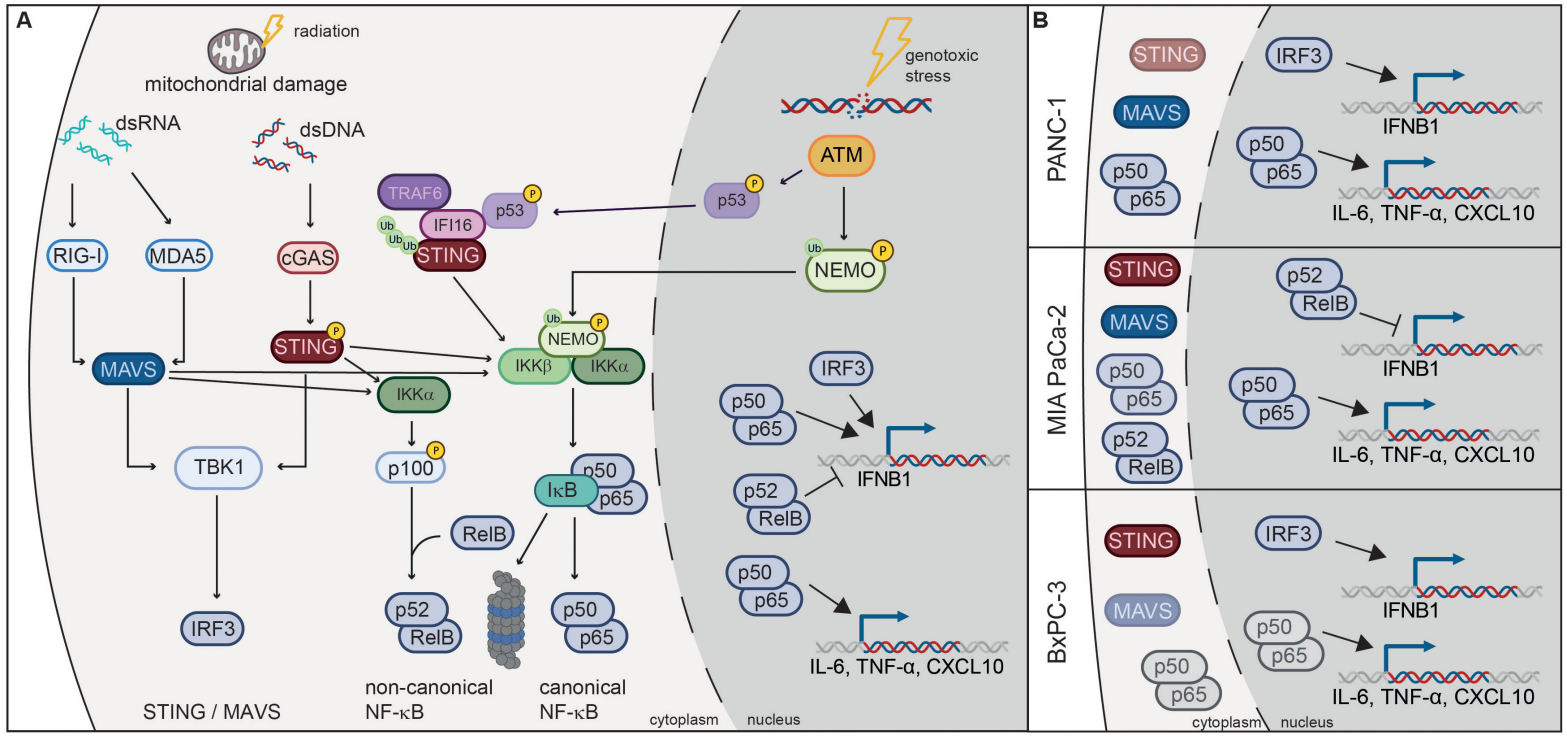
Radiation-induced immunogenic signalling in PDAC cell lines. **(A)** Damaged mitochondria release dsDNA and dsRNA, which activate cGAS/STING and RIG-I/MAVS signalling pathways respectively. Activation of these pathways results in the translocation of transcription factors IRF3 and NF-κB into the nucleus, where they induce the expression of *IFNB1* and proinflammatory cytokines such as *CXCL10*, *IL-6* and *TNF-α*. Activation of the non-canonical NF-κB pathway dampens the induction of *IFNB1*. Canonical NF-κB signalling can also be induced by the ATM kinase, which is activated by double-strand DNA breaks (DSBs) induced by genotoxic stress. ATM phosphorylates NEMO and promotes its monoubiquitination, which is required for translocation into the cytoplasm where it activates the IKK complex. ATM also induces K63-linked ubiquitination of STING through the p53-IFI16-TRAF6 complex, which activates canonical NF-κB. **(B)** STING, MAVS and NF-κB are responsible for radiation-induced expression of *IFNB1* and proinflammatory cytokines in PDAC cell lines. MAVS is the dominant pathway in PANC-1, STING in BxPC-3, while non-canonical NF-κB is induced in MIA PaCa-2 and dampens *IFNB1* expression. NF-κB signalling is more pronounced in PANC-1 and MIA PaCa-2, which harbour oncogenic KRAS mutations G12D and G12C respectively.

The three investigated PDAC cell lines responded similarly to radiation with regard to survival and immunogenic cell death marker secretion but diverged in their ability to activate the IFN-1 axis (**Figure 10**). Despite a strong induction of pSTING and pIRF3, IFNB1 was not detectable in MIA PaCa-2, neither by RT-qPCR nor by ELISA. This may be due to a strong induction of non-canonical NF-κB signalling in irradiated MIA PaCa-2 based on RNA-seq analysis (NFKB2 in **Figure 5H**) and western blot analysis (p100/p52 in **Figure 8A**). Non-canonical NF-κB signalling was previously shown to negatively regulate *IFNB1* expression (44) and may therefore account for the lack of *IFNB1* induction in MIA PaCa-2.

Of the three PDAC cell lines, PANC-1 showed the highest expression of RIG-I/MAVS and the NF-κB subunit RelA (p65), and mounted the strongest IFN-1 response to 3x8 Gy X-rays as evidenced by the induction of pSTAT1, *IFNB1*, *CXCL10* and other ISGs (**Figures 4**, **5H**). Conversely, STING and IRF3 levels were the highest in BxPC-3 (**Figure 4A**). Accordingly, MAVS KO and STING KO showed somewhat stronger reduction in *IFNB1* and *CXCL10* expression in PANC-1 and BxPC-3 cells respectively (**Figures 6**, **7**), suggesting that RIG-I/MAVS is the dominant IFN-1 pathway in PANC-1, while cGAS/STING is more relevant for BxPC-3 cells (**Figure 10B**). Nevertheless, the expression of *IFNB1* and *CXCL10* was strongly reduced in both STING and MAVS KO PANC-1 and BxPC-3 cells, suggesting that cytosolic nucleic acid sensing through both STING and MAVS pathways is essential for the IFN-1 response. Our results also suggest that STING and MAVS act co-dependently as knockout of either of the two genes abrogates *IFNB1* expression. Indeed, STING and MAVS were previously shown to interact and form a complex (52, 53). However, our results are at variance with published data showing no effect of STING depletion on pSTAT1 or *IFNB1* expression in PANC-1 (21, 54).

The high basal activity of NF-κB p65/p50 in PANC-1 cells (**Figure 4**) may be due to the KRAS G12D oncogenic driver mutation, which was shown to induce the expression of IL-1 and thereby activate NF-κB signalling (55). BxPC-3 cells do not have a KRAS mutation, while MIA PaCa-2 harbour the oncogenic G12C mutation. In MIA PaCa-2 phosphorylation of RelA (p65) was induced after 3x8 Gy, but not in BxPC-3 (**Figures 7**, **8**), reinforcing the link between oncogenic KRAS mutations and NF-κB signalling. Radiation-induced expression of *IL-6* and *TNF-α* as NF-κB target cytokines was partly dependent on STING and MAVS in all three PDAC cell lines (**Figure 10B**), which is concordant with previous studies showing that STING and MAVS can activate NF-κB signalling (56–59). Apart from cytosolic nucleic acid sensing pathways, NF-κB can be activated after genotoxic stress by ATM signalling of double-strand DNA breaks (DSBs), resulting in NEMO phosphorylation in the nucleus, followed by its monoubiquitination and export into the cytoplasm where it activates the IKK complex (60). Alternatively, ATM and PARP1 were shown to induce the formation of the p53-IFI16-TRAF6 complex, which ubiquitinates STING and promotes STING-dependent NF-κB activation (61) (**Figure 10A**).

In addition to immunostimulatory effects, radiation-induced IFN-1 response may also upregulate T-cell inhibitory ligands such as PD-L1 and Gal-1. Gal-1 secretion was significantly upregulated following 3x8 Gy at 24 h post irradiation in all three cell lines and at 72 h post irradiation in MIA PaCa-2 and BxPC-3. However, we did not observe radiation-induced PD-L1 expression changes, neither in the two cell lines with no baseline PD-L1 expression (PANC-1 and MIA PaCa-2), nor in the cell line with baseline PD-L1 expression (BxPC-3). Similarly, MHC-I expression was not affected in any of the three investigated cell lines (**Figure 7B**), thus allowing unhindered antigen presentation to T-cells. Therefore, our results suggest that radiation as a means of stimulating anti-tumour immune activation may be considered for PDAC, and Gal-1 specific inhibitors may be of synergistic therapeutic benefit.

The activation of cGAS/STING signalling via STING agonists such as ADU-V16, ADU-S100 and DMXAA was shown to boost immunotherapy approaches in murine models of PDAC by stimulating the production of IFN-regulated chemokines, by promoting infiltration of cytotoxic T-cells and repolarization of M2 immunosuppressive into M1 pro-inflammatory macrophages, and by decreasing the levels of regulatory T-cells (Tregs) (62–64). STING agonists were also shown to enhance the efficacy of radiotherapy by enhancing anti-tumour immunity in PDAC. However, STING agonists act systemically and may induce adverse effects by promoting apoptosis of T-cells (65). Tumour cell-specific activation of cGAS/STING through accumulation of cytosolic dsDNA due to radiation-induced genomic instability of cancer cells may therefore prove advantageous compared to STING agonists. We furthermore demonstrate that protons have similar immunomodulatory effects compared to photons for single doses, supporting the use of particle therapy for PDAC treatment, based on the inverted depth-dose profile that would allow precise, tumour-conformal damage induction and STING axis activation with less risk for healthy surrounding organs.

Moreover, combinatorial treatments with DNA damage response (DDR) inhibitors may sensitize cancer cells to radiation treatment by activating the cGAS/STING and RIG-I/MAVS pathways. Indeed, a previous study showed that ATM inhibition can induce IFN signalling in PDAC cells (PANC-1 and Capan-1) in cGAS/STING-independent but TBK1-dependent manner, which is potentiated by radiation and sensitizes PDAC cells to ICB treatment (66). Further studies will reveal whether other DDR inhibitors can synergize with radiation to induce anti-tumour immunity in PDAC.

## 4 Methods and materials

### 4.1 Cell culture

The human pancreatic cancer cell lines PANC-1, MIA PaCa-2 and BxPc-3 were purchased from ATCC. PANC-1 and MIA PaCa-2 have KRAS mutations (G12D in PANC-1 and G12C in MIA PaCa-2) and are classified as mesenchymal, whereas BxPC-3 is classified as classical. PANC-1 and MIA PaCa-2 were cultured in Dulbecco’s Modified Eagle Medium (DMEM) medium (Gibco, 41965-039), supplemented with 10 % fetal bovine serum (FBS) (Sigma, F7524-500 ml) and 1% penicillin/streptomycin (P/S) (Gibco, 15140-122). BxPC-3 were cultured in Roswell Park Memorial Institute (RPMI) 1640 medium (Gibco, 21875-034), supplemented with 10 % FBS, 1% P/S and 1 % L-Glutamine (Gibco, 25030-024). All cell lines were maintained at 37°C in a humidified atmosphere with 95 % air and 5 % CO_2_. Cells were grown to 70-80 % of confluency at time of irradiation. Prior to irradiation, cell culture flasks were filled air-bubble free with unsupplemented medium to accommodate the vertical flask positioning in both irradiation workflows, which was replaced immediately after irradiation with fresh, supplemented medium.

### 4.2 Cell line generation

To generate CRISPR/Cas9 STING and MAVS KOs, gRNAs targeting exon 3 and 5 respectively were cloned between BbsI sites under the U6 promoter in plasmids encoding Cas9-EGFP or Cas9-Cherry (pX458). gRNA sequences were 5′-CACTGCAGAGATCTCAGCTG-3′ for STING and 5′-GTAGATACAACTGACCCTGT-3′ for MAVS. After 72 h of transfection with polyethylenimine (PEI; Polysciences), GFP- and mCherry-positive BxPc-3 or PANC-1 cells were FACS-sorted and allowed to recover in culture for several days. Cells were subsequently FACS-sorted and GFP- and mCherry-negative cells were seeded 1 cell/well in 96-wells plates. After 14 to 20 days, surviving clones were expanded in culture, genomic DNA was isolated and PCR-amplified Cas9 target region was sequenced. Positive clones were validated by western blotting.

### 4.3 RNA interference

Small interfering RNA (siRNA) oligonucleotides targeting STING as well as control siRNA were purchased from Dharmacon (ON-TARGETplus SMARTpool). 8 x 10^5^ cells (all three cell lines) were seeded in T25 cell culture flasks one day before transfection. Cells were transfected with 150 pmol STING siRNA using Lipofectamine RNAiMax (Invitrogen, 13778-150) and Opti-Mem (Gibco, 31985-062) media. 24 h after transfection cells were irradiated with 8 Gy on three consecutive days (3x8 Gy) and harvested either 24 or 72 h after the last irradiation. Non-irradiated samples were used as a control (0 Gy). Samples were then used for western blotting and RT-qPCR.

### 4.4 Interferon gamma and inhibitor treatments

Recombinant human IFNγ protein (R&D Systems, 285-IF-100) was added at a concentration of 50 ng/µl to the cell cultures 24 h before cell harvest. The STING inhibitor (InvivoGen, H-151) and the NF-κB inhibitor (Cayman Chemical, BI-605906) were both used at a 10 µM concentration continuously 1 h before irradiation until 72 h after the last irradiation.

### 4.5 Irradiation procedures

X-ray irradiations were administered using a horizontal irradiation cabinet (YXLON, TU32-D03, 20 mA, 5.5FOC, filtration: 3 mm Be + 3 mm Al + 0.5 mm Cu), proton irradiations were all performed using the particle synchrotron at MedAustron. For both X-ray and proton irradiation, dedicated irradiation set-ups for all flask types used (Nunc™ Lab-Tek™ ChamberFlasks (170920), T25 (169900) and T75 (156800), all Thermo Fisher) were developed to accommodate for the horizontal beam geometry and ensure standardized positioning. Comprehensive dosimetric verification of all set-ups was performed prior to experiments (**Supplementary Material and Methods, Supplementary Figure 7**), according to recent recommendations for particle beam therapy (67, 68). Cells were irradiated with 0 Gy, 2 Gy, 8 Gy and 24 Gy of physical dose 200 kV X-rays or protons. Additionally, a fractionation regimen of 3x8 Gy was implemented for the X-ray experiments but not for protons due to limited beam time availability.

### 4.6 Clonogenic survival assay

Clonogenic survival assays were performed after 200 kV X-rays or proton irradiations. 2.5 x 10^5^ cells were seeded in Nunc™ Lab-Tek™ ChamberFlasks (170920) one day prior to irradiation. Cells were harvested immediately after irradiation, diluted with supplemented medium appropriate for the cell line and seeded on 6-well plates in previously established, cell line specific concentrations. Following an incubation period of 12 days, cells were fixed with 96% methanol and stained with 0.5% crystal violet solution. Colonies of more than 50 cells were regarded as surviving clones. The linear-quadratic (LQ) formalism was used for survival curve fitting. Surviving fractions in relation to the plating efficiency of non-irradiated control samples were calculated for each value of the delivered physical dose in Gy. The mean and standard deviation result from a minimum of 4 independent experiments. Standard errors result from error propagation. A 1/σ-weighted minimum chi-square estimation was applied to the linear-quadratic model for survival curve fitting. Proton-relative biological effectiveness was calculated for the physical doses that reduced cell survival to 10% (RBE_10_).

### 4.7 Cytokinesis-block micronucleus assay

7 x 10^5^ cells were seeded in Nunc™ Lab-Tek™ ChamberFlasks (170920) one day prior to irradiation. 3 µg/ml cytochalasin B (ThermoFisher, 228090010) was added 48 h after irradiation and after 24 h cells were fixed in 4% paraformaldehyde (Carl Roth), mounted with DAPI-containing mounting medium (VectaShield, H-2000) and evaluated using fluorescence microscopy. Binucleated cells were used for micronuclei quantification.

### 4.8 Western blotting

8 x 10^5^ (PANC-1 and BxPC-3) and 1.2 x 10^6^ cells (BxPC-3) were seeded in T25 flasks one day prior to irradiation. Cells were harvested and lysed with a lysis buffer containing NP40 buffer (Invitrogen, J60766-AP), 1% of protease and phosphatase inhibitor cocktail (Thermo Fisher Scientific, 78440), 1 mM PMSF (Thermo Fisher Scientific, 78431) in DMSO (Thermo Fisher Scientific, D12345) and 1X PhosStop (Merck, 4906837001). The protein concentration was determined with a protein assay kit (Pierce, 10678484). 30 µg of total protein for each sample were separated using SDS-PAGE gels. Proteins were transferred overnight onto PVDF membranes. The membranes were blocked in 5% non-fat milk (Roth, T145.1) for 1 h at room temperature and subsequently incubated with the primary antibody diluted 1:1000 in 5% non-fat milk over night at 4 °C. Washing steps were performed with TBST and membranes were probed with the horseradish peroxidase-conjugated secondary anti-rabbit antibody (7074S, Cell Signaling) for 2 h at room temperature. Membranes were incubated with ECL detection reagents and bands were visualized with ChemiDoc gel imager (Bio-Rad) and quantified using ImageLab software (Bio-Rad). All antibodies are listed in **Supplementary Table 1**.

### 4.9 Subcellular fractionation and whole-cell DNA extraction for mtDNA quantification

Non-irradiated (0 Gy) and 3x8 Gy irradiated cells harvested at 24 h and 72 h after the last irradiation were used for subcellular fractionation followed by isolation of the cytosolic fraction according to Sato et al, 2024 (50). 1 million cells were lysed in digitonin buffer (25 µg/ml digitonin, 150 mM NaCl, 50 mM Hepes, pH 7.4) for 10 min on ice. Lysed samples were centrifuged at 2000 g for 10 min (4 °C). The supernatants were transferred into new tubes and centrifuged at 2000 g for 20 min (4 °C). The two centrifugation steps were repeated three more times. The supernatant after the final centrifugation was used for qPCR-mediated detection of mitochondrial genes. This fractionation procedure does not allow separation of mitochondria from the rest of the cytoplasm. Whole-cell DNA was extracted from 1 million cells using the QIAmp DNA Mini kit (QIAGEN), following the manufacturer´s instructions. An intergenic region on chromosome 5 was analysed in addition to mitochondrial genes and is referred to as genomic DNA (gDNA). Primer sequences are listed in **Supplementary Table 1**.

### 4.10 Real-time quantitative PCR

RNA was extracted using RNeasy Plus extraction kit (Qiagen, 74136) according to the manufacturer’s instructions. Residual gDNA removal and subsequent cDNA synthesis was performed using iScript™ gDNA Clear cDNA Synthesis Kit (Bio-Rad, 1725034) according to the manufacturer’s instructions. *IFNB1*, *CXCL10*, *IL-6* and *TNF-α* levels relative to *TBP* were quantified by multiplexing RT-PCR based on commercial primer sets (human *IFNB1* UniqueAssayID: qHsaCEP0054112, human *CXCL10* UniqueAssayID: qHsaCEP0053880, human *IL-6* UniqueAssayID: qHsaCEP0051939, human *TNF* UniqueAssayID: qHsaCEP0040184, human *TBP* UniqueAssayID: qHsaCIP0036255). PCRs were run on a CFX Opus machine (Bio-Rad) operated by the embedded CFX Maestro software (Bio-Rad) using iQ™ Multiplex Powermix (Bio-Rad). RT-PCR data were analzyed using the ΔΔCt method with TBP as a housekeeping gene.

### 4.11 RNA isolation and RNA-seq library preparation

Cells were harvested and counted, 8 million PDAC cells were mixed with 2 million Drosophila S2 cells as a spike-in control. Cell pellets were resuspended in 1 ml TRI reagent (Sigma). 200 µL chloroform (Applichem) was added, samples were mixed and centrifuged at 4 °C maximum speed for 15 min. The upper phase was transferred to a new tube and subjected to isopropanol precipitation. 20 µg of RNA were treated with 40 U DNase I (Roche, 50-100-3290) at 37 °C for 30 min and purified by phenol-chloroform extraction and ethanol precipitation. rRNA was depleted and RNA-seq libraries were prepared with NEBNext rRNA depletion kit v2 (Human/Mouse/Rat) and NEBNext Ultra II Directional RNA Library Prep Kit for Illumina (E7760S) according to the manufacturer’s instructions using 600 ng total RNA input. Sequencing was performed on an Illumina NovaSeq 6000 instrument in readmode SR100 by the Next Generation Sequencing facility at Vienna BioCenter Core Facilities (VBCF).

### 4.12 RNA-seq data analysis

RNA-seq data from HEK293 cells from different genetic background was processed using PiGx-RNA-seqc pipeline (69). In short, the data was quantified using the GRCh38/hg38, and the dm6 versions of the human and Drosophila spike-in transcriptome (downloaded from the ENSEMBL database (70) using SALMON) (71) with default parameters. For visualization purposes, the data was mapped to the GRCh38/hg38, and dm6 versions of the human, and Drosophila genomes using STAR, with the following parameters: –limitOutSJcollapsed 20000000 –limitIObufferSize=1500000000 –outFilterMultimapNmax 10 –seedPerWindowNmax 5. The quantified data was processed using tximport (72) and the differential expression analysis was done using DESeq2 (73). Genes with less than 5 reads in all biological replicates of one condition were filtered out before the differential analysis. The data was normalized by taking the ratio of reads mapping to the human and the Drosophila transcriptome. Genes were defined as differentially expressed if they had a minimum absolute log2 fold change of 1, and a BH adjusted p value less than 0.05. Genes from Hallmark gene sets from Molecular Signatures Database (MSigDB) (74) were tested for enrichment among differentially expressed genes using Gene set enrichment analysis (GSEA) algorithm (75) implemented in clusterProfiler R package (76). Gene sets with adjusted p-value < 0.05 were defined as enriched. RNA-seq data have been deposited in ArrayExpress under accession code: E-MTAB-13096.

### 4.13 ELISA

2.5 x 10^5^ cells (all cell lines) were seeded in T25 flasks one day prior to irradiation. Galectin-1, HMGB1 and IFNB1 levels in cell culture supernatants were quantified with ELISA kits (Galectin-1: DGAL10, R&D Systems; HMGB1: ST51011, IBL International; IFNB1: 41415-1, PBL Assay Science) as per the manufacturer´s recommendations. Photometric assessment was performed on a Varioskan Flash Microplate Reader (Thermo Scientific) using SkanIt Software 2.4.5 (Thermo Scientific).

### 4.14 Immunofluorescence microscopy

For immunofluorescence analyses, 1 x 10^5^ cells (all cell lines) were seeded in Nunc™ Lab-Tek™ ChamberFlasks one day prior to irradiation treatment. Immunofluorescence experiments were performed 24 h post irradiation. Cells were fixed in 4% paraformaldehyde (Carl Roth), permeabilized with 0.1 % Tween20 (Sigma Aldrich) and 0.01 % Triton X-100 (Sigma Aldrich) in PBS and blocked with 1% BSA (Sigma Aldrich). Primary antibodies targeting dsDNA (Abcam, ab27156, 1:1000 dilution), dsRNA (Cell Signaling, 28764, 1:1000 dilution) or TFAM (Genetex, GTX103231, 1:500 dilution) were applied over night at 4 °C, followed by Rhodamine (TRITC)-conjugated AffiniPure goat anti-mouse IgG (Jackson ImmunoResearch, 115-025-072, 1:400 dilution) or Alexa Fluor 488-conjugated goat anti-rabbit IgG (Abcam, ab150077, 1:400 dilution) for 1 h at room temperature. Cells were mounted with DAPI-containing mounting medium (VectaShield, H-2000) and evaluated using fluorescence microscopy. Images were acquired with an Axio Z2 fluorescence microscope (Zeiss) or the LSM980 inverse confocal microscope (Zeiss) equipped with a Plan-Apochromat 63x/1.4 Oil DIC (WD 0.13 mm), a 405 nm laser diode (30mW), a 488 nm laser diode (30mW) and a 561 nm DPSS laser (25mW). Appropriate laser power, excitation wavelength range and pinhole size were chosen for each channel in alignment with negative controls. Images were acquired with Zen software (version 3.3, Zeiss) and processed with Fiji (ImageJ). The threshold was set to 15000 (dsDNA) and 10000 (TFAM). Fluorescence and co-localization signals were analysed with the “Analyze Particles” option in Fiji. For co-localization the number of overlapping objects with specifications of 0.1-infinity/0.1-1.0 (TFAM) and 0.05-1/0.2-1.0 (dsDNA) size/circularity were counted. Micronuclei were assessed using DAPI nuclear counterstain in the same samples used for dsDNA evaluation.

### 4.15 Flow cytometry

1.5 x 10^6^ cells (PANC-1 and BxPC-3) or 2 x 10^6^ (BxPC-3) were seeded in T75 flasks one day prior to irradiation. Cells were harvested with 0.5 mM EDTA and then stained for surface markers using fluorochrome-conjugated antibodies against HLA-A,B,C-APC-Cy7 (Biolegend, 311426) or PD-L1-APC (Biolegend, 329708). As positive control antibody, CD59-FITC (BD Pharmingen, 555763) was used. 5 x 10^5^ cells in 50 µl were incubated with optimal amounts of diluted antibodies (20 µl) on ice for 30 minutes. Subsequentially, cells were washed with PBS+0.5 % BSA+0.05 % NaN_3_ and 10 µl of 1:4000 diluted DAPI was added. Samples were acquired on Cytoflex S flow cytometer (Beckmann Coulter, Brea, CA) and results were analyzed using the FlowJo™ software (BD Biosciences). Live cells were distinguished according to their forward- and side scatter characteristics and as being DAPI negative. Positivity was confirmed by isotype control antibodies and fluorescence minus one controls. Treatment-induced autofluorescence was accounted for by correcting the mean fluorescence intensity (MFI) of antibody-stained cells for the mean fluorescence of unstained cells for each individual sample.

### 4.16 Statistical analysis

All experiments were performed in 3-5 independent biological replicates and statistical significance was determined using two-way analysis of variance (ANOVA) with multiple comparisons correction: *≤ 0.05; **≤ 0.01; ***≤ 0.005, ****≤0.0001.

## Data Availability

The datasets presented in this study can be found in online repositories. The names of the repository/repositories and accession number(s) can be found below: ArrayExpress under accession code: E-MTAB-13096.
