# Supplementary material for: Cytosolic nucleic acid sensors and interferon beta-1 activation drive radiation-induced anti-tumour immune effects in human pancreatic cancer cells
Source: Front Immunol. 2024 Sep 20;15:1286942. doi: 10.3389/fimmu.2024.1286942 (PMC11449851; doi:10.3389/fimmu.2024.1286942)
Supplement: Supplementary file 1 [file DataSheet1.docx]

Supplementary Material

# Supplementary Materials and Methods

**1.1 Physical parameters and dosimetric details of the experimental irradiation set-up**

We provide physical parameters of our particle beams for pre-clinical research in accordance with the report of a National Cancer Institute special panel (1). Reduced uncertainties in physical factors will pave the way for best possible biological outcomes. The radiation delivery technique was pencil beam scanning and no ranger shifters or ripple filters were used to modify the proton beam. The proton treatment plan was designed for a field size of 17x9 cm², with a spread-out Bragg peak (SOBP) of 4 cm and a nominal energy of 124.7 MeV (range at 80 % dose level in water = 101.2 mm). The treatment planning system RayStation v7.99 (RaySearch Laboratories, Stockholm, Sweden) employing a Monte Carlo based dose calculation algorithm (v4.3) was utilized. The physical dose was delivered with a particle rate of about 8·10⁸ particles/s. The energy layers were spaced either 1 mm or 2 mm apart.

Customized holders for radiobiological cell irradiations were developed and commissioned in-house for use in light ion beams. Their dosimetric characterization (Supplementary Figure 7) was performed using a plane-parallel ionization chamber (Advanced Markus® Electron Chamber, PTW, Freiburg, Germany) as well as a cylindrical ionization chamber (0.125 cc Semiflex Chamber, PTW, Freiburg, Germany). Repeated ionization chamber response measurements were carried out along the entire depth dose curve, resulting in a reproducibility of 0.55% expressed as a standard deviation. The general positioning uncertainty was assessed by taking into account the reproducibility, resolution of the water phantom scale and positioning of both ionization chambers (2).

Dosimetric validation in terms of the physical absorbed dose as well as the microdosimetric parameter dose averaged linear energy transfer (LETd) was performed using the GATE Monte Carlo platform, which is based on the Geant4 toolkit. The treatment plan used for cell irradiation was converted into a particle source description at vacuum exit which was used as input for the validated beam model of our nozzle design (3). A simple scoring geometry was chosen: A cylinder with a radius of 2.5 mm, corresponding to the collecting electrode dimensions of the Advanced Markus ionization chamber, and a height of 150 mm. The DoseActor and LETActor (both are standard GATE packages) were attached to this scoring cylinder, with a voxel size along the height of the cylinder of 0.75 mm each. The physics list chosen was QGSP_INCLXX_EMZ and the number of primary particles was 10⁸ over the entire plan. For the LETd simulations, only primary protons were scored.

# Supplementary Table 1

| REAGENT or RESOURCE | SOURCE | | IDENTIFIER | | |
| --- | --- | --- | --- | --- | --- |
| Antibodies | | | | |  |
| Rabbit anti-β-Actin | Cell Signaling | | RRID: AB_2223172; 4970S; 1:1000 for WB | | |
| Rabbit anti-GAPDH | Cell Signaling | | RRID: AB_10622025; 5174S; 1:1000 for WB | | |
| Rabbit anti-cGAS | Cell Signaling | | RRID: AB_2732795; 15102S; 1:1000 for WB | | |
| Rabbit anti-pSTING | Cell Signaling | | RRID: AB_2737062; 19781S; 1:1000 for WB | | |
| Rabbit anti-STING | Cell Signaling | | RRID: AB_2732796; 13647S; 1:1000 for WB | | |
| Rabbit anti-RIG-I | Cell Signaling | | RRID:AB_2269233; 3743S; 1:1000 for WB | | |
| Rabbit anti-MAVS | Cell Signaling | | RRID:AB_2798889; 24930S; 1:1000 for WB | | |
| Rabbit anti-pTBK1 | Cell Signaling | | RRID: AB_10693472; 5483S; 1:1000 for WB | | |
| Rabbit anti-TBK1 | Cell Signaling | | RRID: AB_2827657; 38066S; 1:1000 for WB | | |
| Rabbit anti-pIRF3 | Cell Signaling | | RRID: AB_823547; 4947S; 1:1000 for WB | | |
| Rabbit anti-IRF3 | Cell Signaling | | RRID: AB_1904036; 4302S; 1:1000 for WB | | |
| Rabbit anti-phospho-IKKα/β (Ser176/180) | Cell Signaling | | RRID:AB_207938; 2697S; 1:1000 for WB | | |
| Rabbit anti-IKKβ | Cell Signaling | | RRID:AB_11024092; 8943S; 1:1000 for WB | | |
| Rabbit anti-Phospho-NF-κB p65 (Ser536) | Cell Signaling | | RRID:AB_331284; 3033S; 1:1000 for WB | | |
| Rabbit anti-NF-κB p65 | Proteintech | | RRID:AB_2178878; 10745-1-AP1;1000 for WB | | |
| Rabbit anti-NF-κB2 p100/p52 | Cell Signaling | | RRID:AB_10695537; 4882S; 1:1000 for WB | | |
| Rabbit anti-TYK2 | Cell Signaling | | RRID:AB_2798419; 14193S; 1:1000 for WB | | |
| Rabbit anti-phospho-TYK2 | Cell Signaling | | RRID:AB_2303972; 9321S; 1:1000 for WB | | |
| Rabbit anti-pSTAT1 | Cell Signaling | | RRID: AB_561284; 9167S; 1:1000 for WB | | |
| Rabbit anti-STAT1 | Cell Signaling | | RRID: AB_2737027; 14994S; 1:1000 for WB | | |
| Rabbit anti-pSTAT3 | Cell Signaling | | RRID:AB_2491009; 9145S; 1:1000 for WB | | |
| Mouse anti-STAT3 | Abcam | | RRID:AB_10901752; Ab119352; 1:1000 for WB | | |
| Rabbit anti-PD-L1 | Cell Signaling | | RRID AB_2687655; 13684T; 1:1000 for WB | | |
| Anti-rabbit IgG, HRP-linked Antibody | Cell Signaling | | RRID: AB_2099233; 7074S; 1:1000 for WB | | |
| Mouse anti-dsDNA | Abcam | | RRID: AB_470907; ab27156; 1:1000 for IF | | |
| Mouse anti-dsRNA | Cell Signaling | | 28764; 1:1000 for IF | | |
| Rabbit anti-TFAM | Genetex | | RRID: AB_11176720; GTX103231; 1:500 for IF | | |
| Rhodamine (TRITC) AffiniPure Goat Anti-Mouse IgG, F(ab')₂ fragment specific | Jackson Immunoresearch | | RRID: AB_2338486; 115-025-072; 1:400 for IF | | |
| Goat Anti-Rabbit IgG H&L (Alexa Fluor® 488) | Abcam | | RRID: AB_2630356; ab150077; 1:400 for IF | | |
| APC/Cyanine7 anti-human HLA-A,B,C Antibody | Biolegend | | 311426 | | |
| APC anti-human CD274 (B7-H1, PD-L1) Antibody | Biolegend | | RRID: AB_940360; 329708 | | |
| FITC Mouse Anti-Human CD59 | BD Pharmingen | | 555763 | | |
| siRNAs | | | | |  |
| ON-TARGETplus SMARTpool TMEM173 (STING): | Dharmacon |  | |  |  |
| 5’-GGUCAUAUUACAUCGGAUA-3’ |  | J-024333-05 | |  |  |
| 5’-AACAUUCGCUUCCUGGAUA-3’ |  | J-024333-06 | |  |  |
| 5’-GCAUCAAGGAUCGGGUUUA-3’ |  | J-024333-07 | |  |  |
| 5’-GCACCUGUGUCCUGGAGUA-3’ |  | J-024333-08 | |  |  |
| primers | | | | |  |
| \| MT-ND1-fwd: GGCTATATACAACTACGCAAAGGC \| \| --- \| \| MT-ND1 rev: GGTAGATGTGGCGGGTTTTAGG  MT-ND2-fwd: CTTCTGAGTCCCAGAGGTTACC \| \| MT-ND2-rev: GAGAGTGAGGAGAAGGCTTACG \| \| untr5_fw CTGTACCTGGGGTTCATTCATT  untr5_rev CAGTAAGCCGTTCACTCTCACA \| | Microsynth |  | |  |  |
| Experimental Models: Cell Lines | | | | |  |
| PANC-1 | ATCC | | RRID: CVCL_0480; CRL-1469 | | |
| MIA PaCa-2 | ATCC | | RRID: CVCL_0428; CRL-1420 | | |
| BxPC-3 | ATCC | | RRID: CVCL_0186; CRL-1687 | | |
| Software and Algorithms | | | | |  |
| ImageLab | Biorad | | http://www.bio-rad.com/de-at/product/image-lab-software?ID=KRE6P5E8Z | | |
| Fiji | NIH | | RRID: SCR_002285 | | |
| FlowJo | BD Biosciences | | RRID: SCR_008520 | | |
| Zen version 3.3 | Zeiss | | RRID: SCR_013672 | | |
| PiGx RNA-seq | Max Delbrück Center for Molecular Medicine | |  | | |
| SALMON | Patro et al. 2017 (37) | | RRID: SCR_017036 | | |
| STAR RNA-seq aligner | Alexander Dobin | | RRID: SCR_004463 | | |
| tximport | Soneson et al. 2016 (38) | | RRID: SCR_016752 | | |
| DESeq2 | Love et al. 2014 (39) | | RRID: SCR_015687 | | |
| ClusterProfiler R package v3.18.1 | Yu et al. 2012 (40) | | RRID: SCR_016884 | | |
| SkanIt Software 2.4.5 | Thermo Scientific | |  | | |
| CFX Maestro | Biorad | | http://www.bio-rad.com/de-at/product/cfx-maestro-software-for-cfx-real-time-pcr-instruments?ID=OKZP7E15 | | |

# Supplementary Figures

**
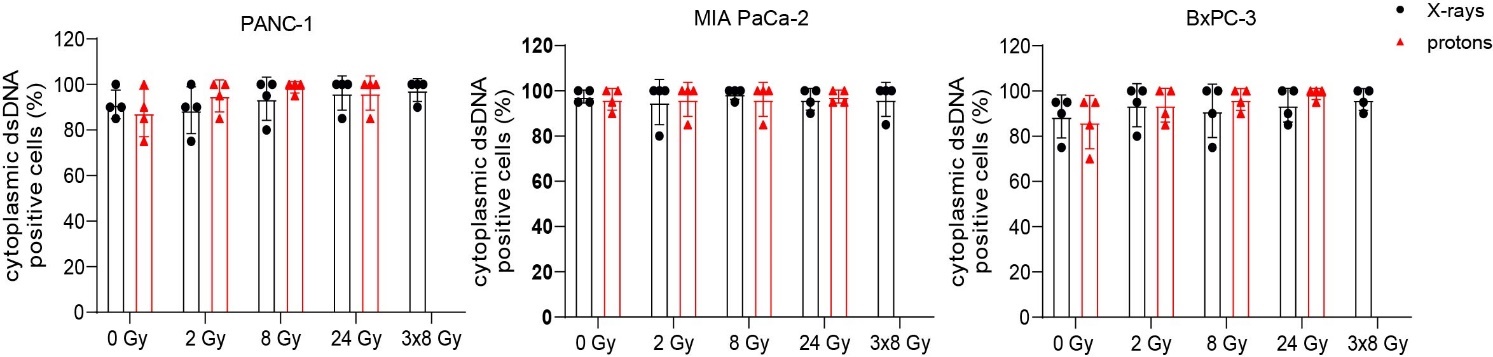
**

**Supplementary Figure 1. Radiation induces cytosolic dsDNA accumulation in human PDAC cell lines.** The percentage of PANC-1, MIA PaCa-2 and BxPC-3 cells positive for cytosolic dsDNA 24 h after radiation with different doses of X-rays (black circles) or protons (red triangles) (N=4). Data points represent mean values ± standard deviation.


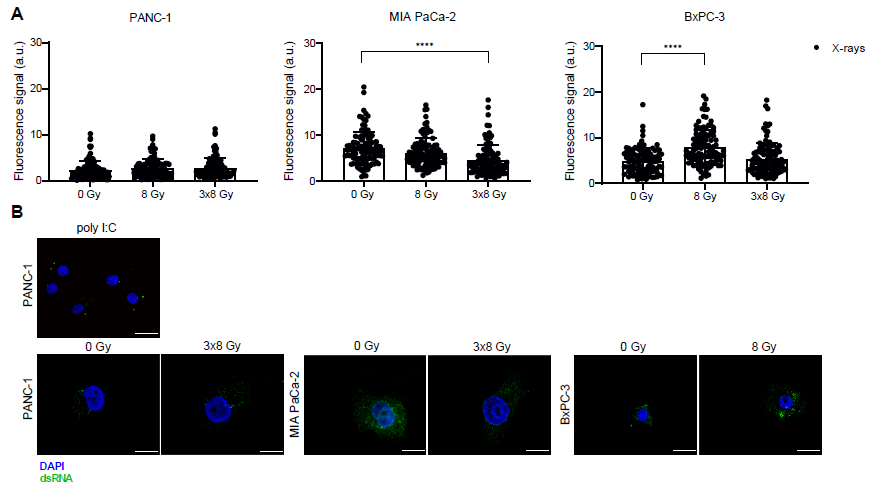


**Supplementary Figure 2. The analysis of dsRNA in irradiated PDAC cells.** (A,B) Cytosolic dsRNA content in PANC-1, MIA PaCa-2 and BxPC-3 cells 3 h after irradiation with different doses of X-rays. The 3 h timepoint was chosen based on a time course experiment showing the strongest signal 3 h after irradiation. Three experiments were performed and 105 cells were analysed per dose group. (A) Quantification and (B) representative images are shown. Poly I:C was used as a positive control for antibody verification. Scale bar = 20 µm.


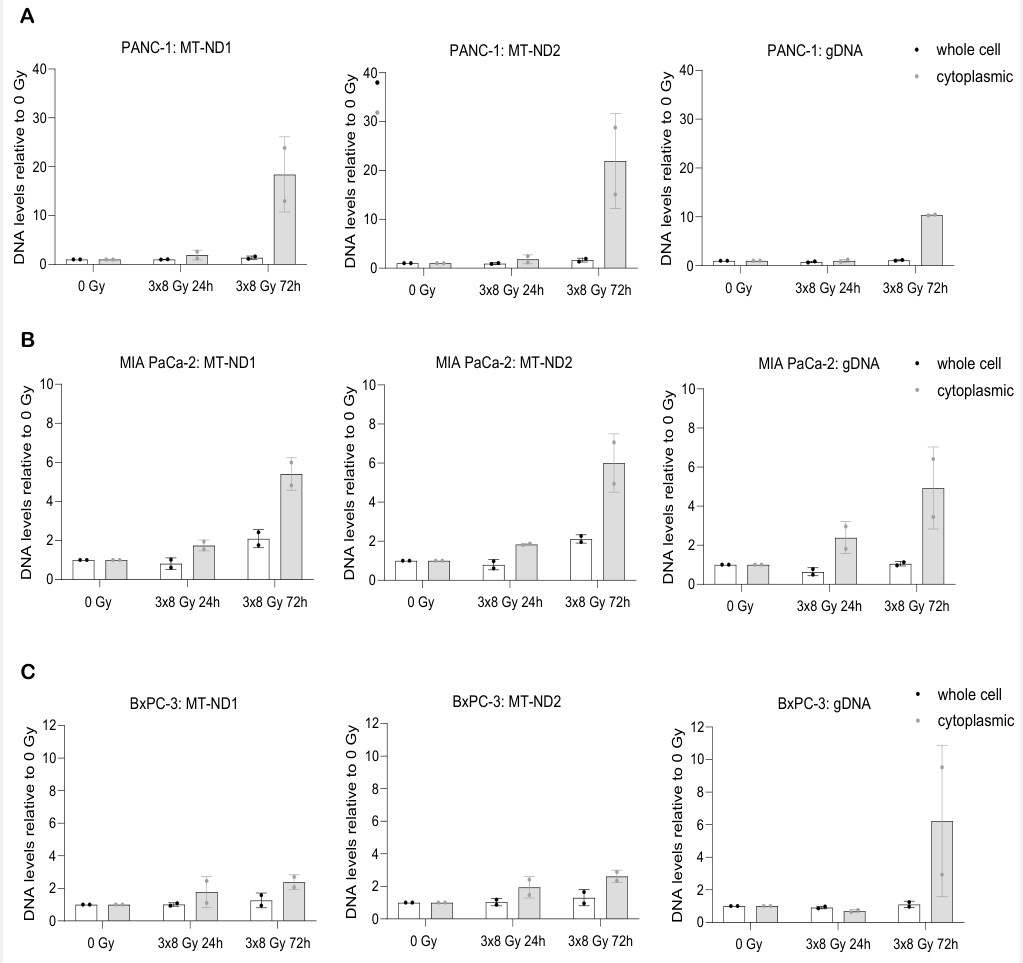


**Supplementary Figure 3.** Radiation-induced cytosolic dsDNA has mitochondrial and genomic origins. Mitochondrial DNA (ND1 and ND2 genes) and genomic DNA contents in whole cell versus cytosolic cell compartments were determined in PANC-1 (A), MIA PaCa-2 (B) and BxPC-3 (C) using subcellular fractionation and qPCR at 24 h and 72 h after 3x8 Gy compared to non-irradiated 0 Gy controls (N=2).


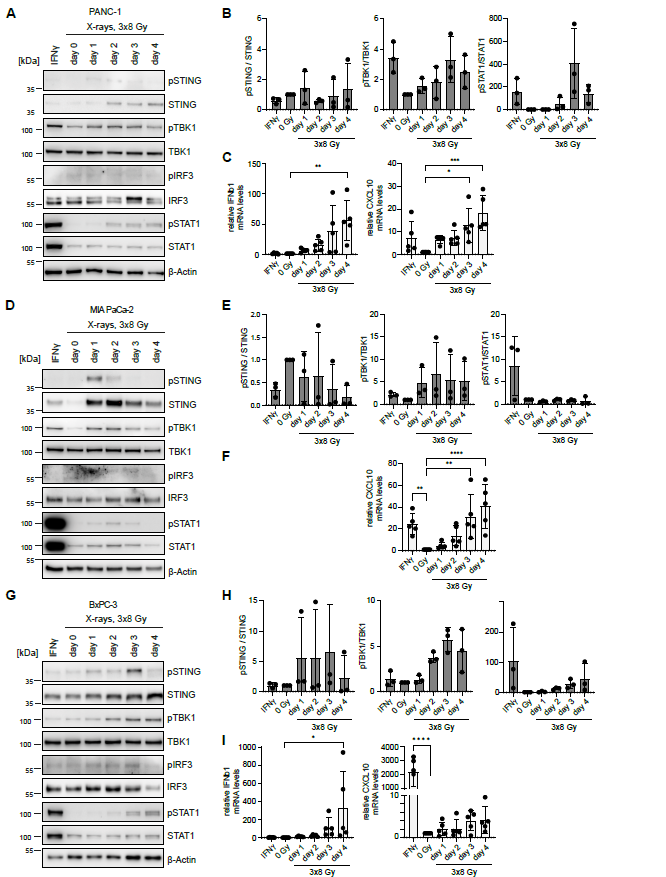


**Supplementary Figure 4. Delayed onset of *IFNB1* activation in human PDAC cells after fractionated X-ray dose.** (A,D,G) Western blot analysis of STING signalling in (A) PANC-1, (D) MIA PaCa-2 and (G) BxPC-3. Cells were either untreated (0 Gy), stimulated with 50 µg/ml IFNγ or irradiated with 3x8 Gy X-rays and harvested 1 to 4 days after radiation. (B,E,H) Relative phosphorylation levels of STING, TBK1 and STAT1 compared to unphosphorylated protein. (C,F,I) Relative mRNA levels of *IFNB1* and *CXCL10* evaluated by RT-qPCR. No qPCR Ct values could be determined for MIA PaCa-2 *IFNB1*. Data points represent mean values of 3 (western blots) or 5 (qPCR) independent experiments ± standard deviation.


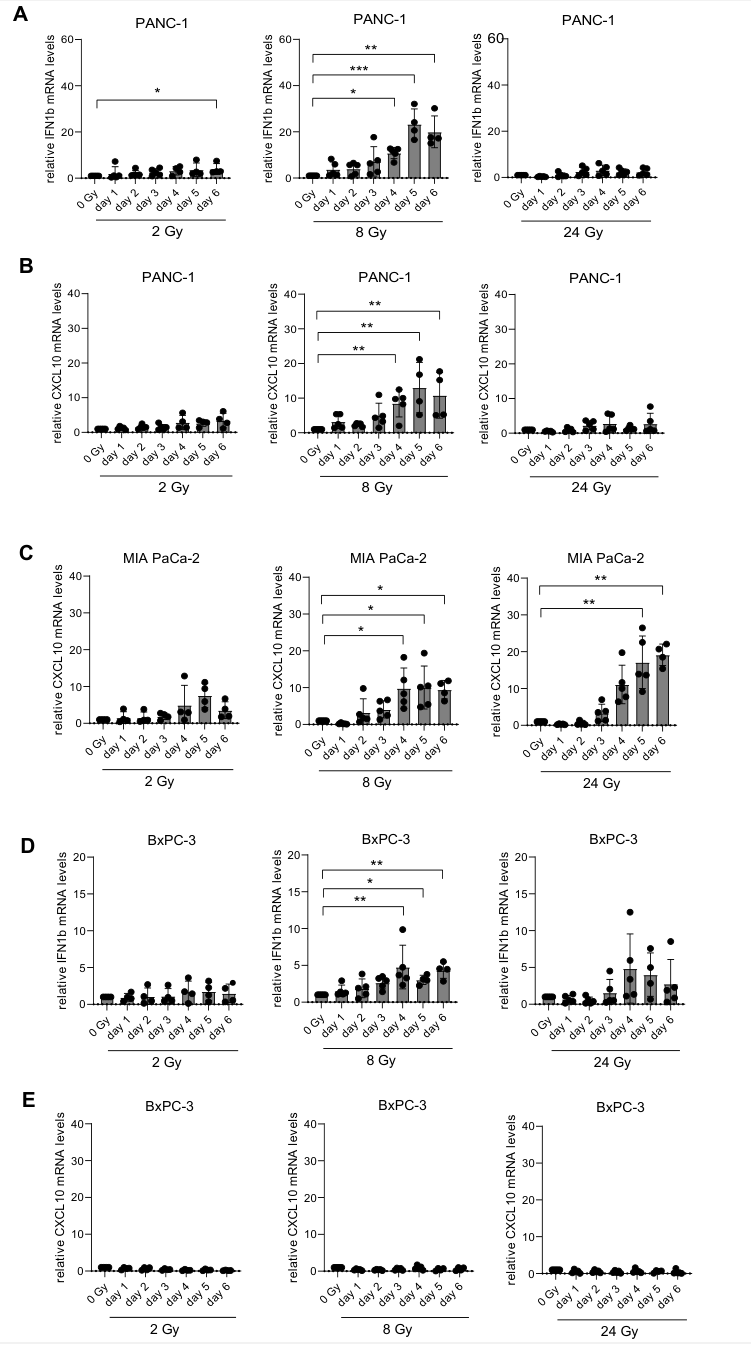


**Supplementary Figure 5. *IFNB1* activation in human PDAC cells after single dose X-ray irradiation.** Relative expression of *IFNB1* and *CXCL10* compared to 0 Gy were determined by RT-qPCR for PANC-1 (A,B), MIA PaCa-2 (C) and BxPC-3 (D,E). No qPCR Ct values could be determined for MIA PaCa-2 *IFNB1*. Data points represent mean values of 4-5 independent experiments ± standard deviation.

**
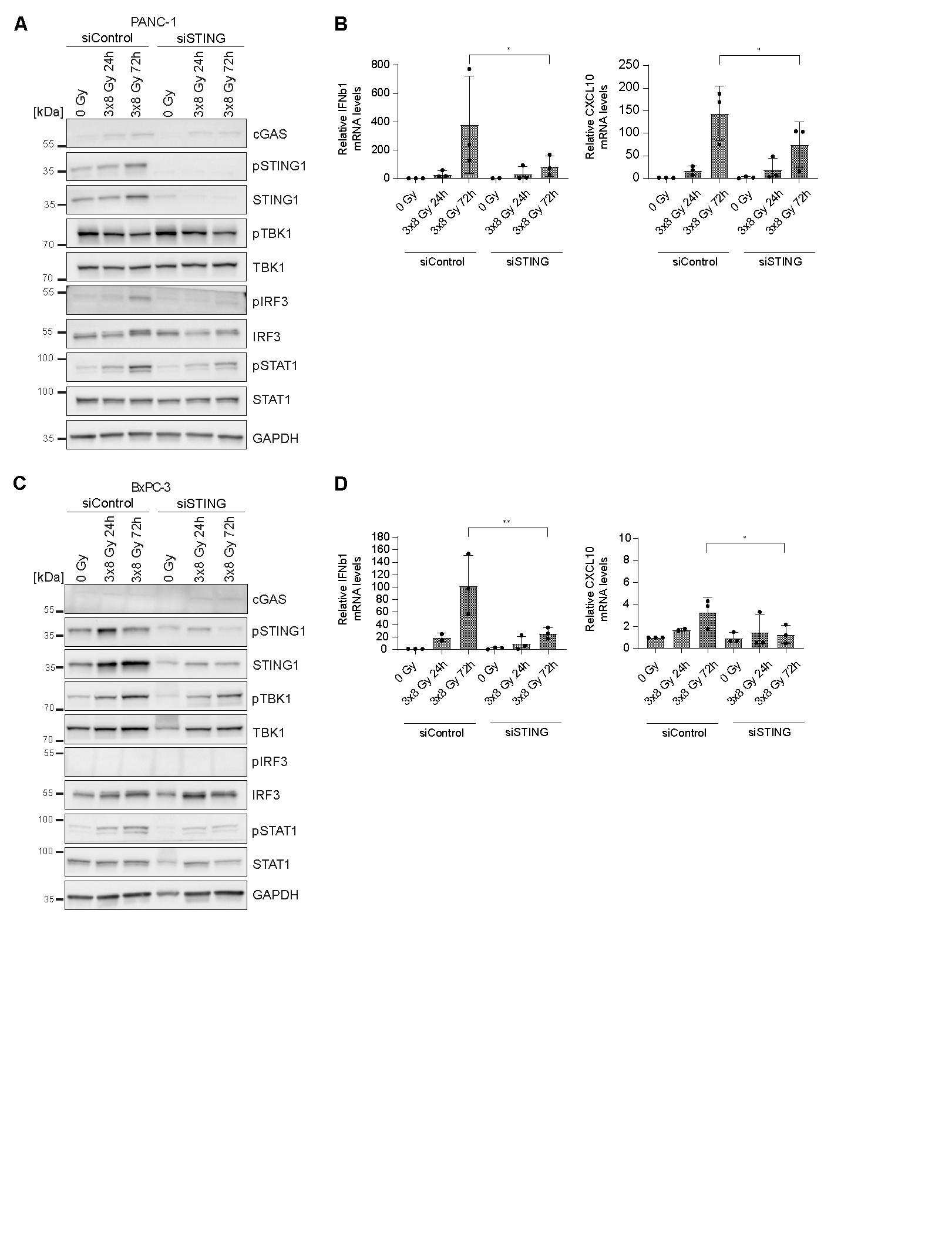
**

**Supplementary Figure 6. Radiation-mediated *IFNB1* activation is STING-dependent in human PDAC cells.** (A,C) Western blot analysis of cGAS/STING signalling after siRNA-mediated STING depletion in (A) PANC-1 and (C) BxPC-3. Cells were either untreated (0 Gy) or irradiated with 3x8 Gy X-rays and harvested 24 h and 72 h after radiation. Cells were transfected with siControl or siSTING 24 h before irradiation. (B,D) Relative mRNA levels of *IFNB1* and *CXCL10* evaluated by RT-qPCR in (B) PANC-1 and (D) BxPC-3. Data points represent the mean values of 3 independent experiments ± standard deviation.

**
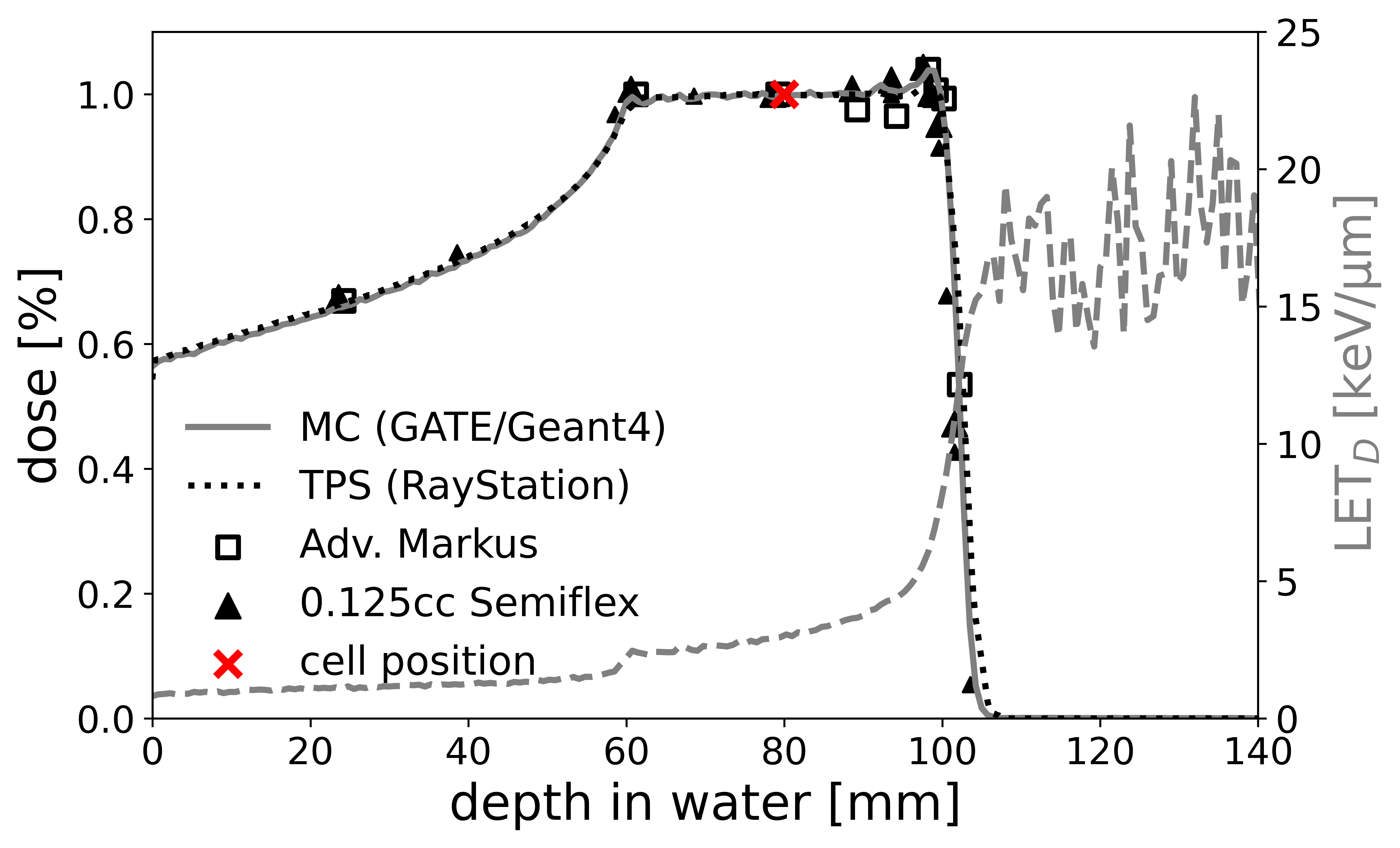
**

**Supplementary Figure 7. Dose and LET_d_ profiles for protons, calculated using the Monte Carlo toolkit GATE v9.0.** Dose measurements are indicated with black squares for Advanced Markus chamber measurements and black triangles for Semiflex ionization chamber measurements. On the mirrored y-axis, the dose-averaged linear energy transfer (LET_d_) of primary protons is shown. Cell flasks were always positioned in the center of the SOBP with a high-precision water phantom scale.

# Supplementary References

1. Durante M, Paganetti H, Pompos A, Kry SF, Wu X, Grosshans DR. Report of a National Cancer Institute special panel: Characterization of the physical parameters of particle beams for biological research. Med Phys. 2019;46(2):e37-e52.

2. Barna S, Resch AF, Puchalska M, Georg D, Palmans H. Technical note: Experimental determination of the effective point of measurement of the PTW-31010 ionization chamber in proton and carbon ion beams. Med Phys. 2022;49(1):675-81.

3. Elia A, Resch AF, Carlino A, Böhlen TT, Fuchs H, Palmans H, et al. A GATE/Geant4 beam model for the MedAustron non-isocentric proton treatment plans quality assurance. Phys Med. 2020;71:115-23.
